# C9orf72 poly-PR forms anisotropic condensates causative of nuclear TDP-43 pathology

**DOI:** 10.1016/j.isci.2024.110937

**Published:** 2024-09-14

**Authors:** Rachel E. Hodgson, Jessica A. Rayment, Wan-Ping Huang, Anna Sanchez Avila, Brittany C.S. Ellis, Ya-Hui Lin, Nikita Soni, Guillaume M. Hautbergue, Tatyana A. Shelkovnikova

**Affiliations:** 1 Sheffield Institute for Translational Neuroscience and Neuroscience Institute, University of Sheffield, Sheffield S10 2HQ, UK

**Keywords:** Biochemistry, Molecular biology, Cell biology

## Abstract

Proteinaceous inclusions formed by *C9orf72*-derived dipeptide-repeat (DPR) proteins are a histopathological hallmark in ∼50% of familial amyotrophic lateral sclerosis/frontotemporal dementia (ALS/FTD) cases. However, DPR aggregation/inclusion formation could not be efficiently recapitulated in cell models for four out of five DPRs. In this study, using optogenetics, we achieved chemical-free poly-PR condensation/aggregation in cultured cells including human motor neurons, with spatial and temporal control. Strikingly, nuclear poly-PR condensates had anisotropic, hollow-center appearance, resembling TDP-43 anisosomes, and their growth was limited by RNA. These condensates induced abnormal TDP-43 granulation in the nucleus without stress response activation. Cytoplasmic poly-PR aggregates forming under prolonged opto-stimulation were more persistent than its nuclear condensates, selectively sequestered TDP-43 in a demixed state and surrounded spontaneous stress granules. Thus, poly-PR condensation accompanied by nuclear TDP-43 dysfunction may constitute an early pathological event in C9-ALS/FTD. Anisosome-type condensates of disease-linked proteins may represent a common molecular species in neurodegenerative disease.

## Highlights

• Optogenetics can be used to model *C9orf72* DPR condensation in neurons
• Opto-PR forms hollow-center nuclear condensates, and RNA limits their growth
• Opto-PR condensation elicits stress-independent nuclear TDP-43 pathology
• Cytoplasmic poly-PR assemblies are persistent and selectively sequester TDP-43

## Graphical abstract

**Figure:**
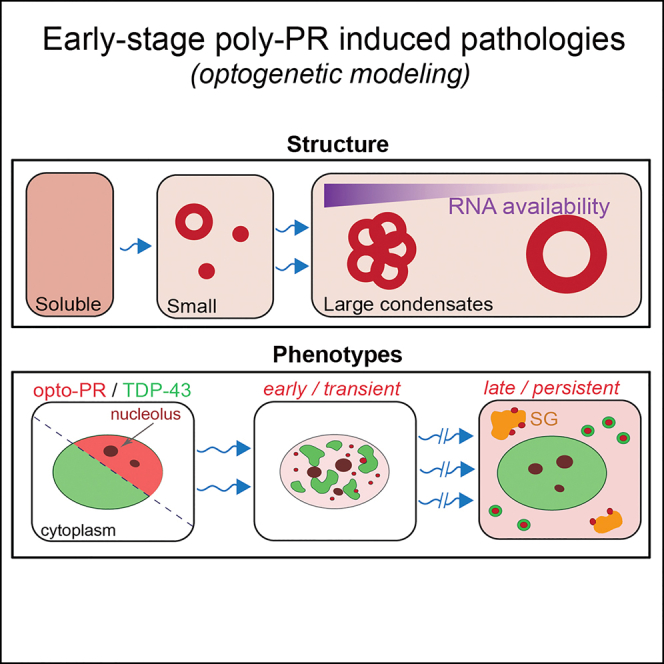

## Introduction

A G_4_C_2_ hexanucleotide repeat expansion (HRE) in the first intron of the *C9orf72* gene is the most common genetic alteration associated with amyotrophic lateral sclerosis (ALS) and frontotemporal dementia (FTD).^1^^,^^2^ Healthy individuals commonly carry 2 repeats, while C9-ALS/FTD is associated with ≥30 repeat lengths.^3^ Production of dipeptide repeat (DPR) proteins from the *C9orf72*-HRE transcripts is one of the proposed mechanisms of repeat toxicity.^4^^,^^5^^,^^6^ Both sense and antisense C9orf72-HRE transcripts are translated in all reading frames via non-canonical, repeat-associated non-AUG (RAN) translation to produce five DPRs: poly-GA, -GR (sense), -PR, -PA (antisense), and -GP (both sense and antisense). All five DPRs have been detected in the patient CNS, primarily as cytoplasmic and less often nuclear inclusions in neurons and glia.^7^^,^^8^^,^^9^^,^^10^^,^^11^ While poly-GA and -GP are the most abundant, arginine containing DPRs (R-DPRs) poly-GR and poly-PR are the most toxic species in cells and *in vivo.*^12^^,^^13^^,^^14^ For example, nuclear poly-PR aggregation was sufficient to induce ALS-like phenotypes in non-human primates^15^ and expression of this DPR was extremely toxic in mice.^16^^,^^17^

R-DPR inclusion pathology seen in patients and mouse models^18^^,^^19^^,^^20^ is not easily reproducible in cultured cells, where R-DPRs typically display diffuse distribution (outside the nucleolus), even when overexpressed and independent of the repeat length; this is in contrast to poly-GA that readily forms large cytoplasmic aggregates.^21^^,^^22^^,^^23^^,^^24^^,^^25^ High arginine content renders R-DPRs highly hydrophilic and hence soluble. Although poly-PR and poly-GR have similar biochemical properties, molecular dynamics simulations revealed that poly-PR is capable of (limited) self-association—forming either dimers or small amorphous oligomers, whereas poly-GR is not likely to form any stable oligomeric species.^26^ Consistently, *in vitro*, R-DPRs undergo phase separation only in the presence of additional agents—RNA or proteins^17^^,^^27^^,^^28^^,^^29^ This suggests that certain factors in the cellular environment drive R-DPR loss of solubility in ALS/FTD. Sensitive immunoassays revealed that soluble DPRs are less abundant in the more affected brain regions, as compared to the relatively spared regions.^30^

Intermediate products of R-DPR aggregation may contribute to cellular dysfunction, however, due to difficulty of modeling this molecular event, their role could not be investigated in the cellular setting. We hypothesized that the use of optogenetics^31^^,^^32^ will allow circumventing the intrinsic solubility of R-DPRs, by triggering and maintaining an oligomerized R-DPR state. Indeed, using Cry2olig tagging, we were able to reliably induce condensation of poly-PR (“opto-PR”) in cultured cells, with spatial and temporal control. Using this model, we show that: (i) Poly-PR can form nuclear condensates with a specific ordered arrangement reminiscent of TDP-43 “anisosomes”,^33^ and these assemblies trigger abnormal nuclear granulation of TDP-43. (ii) Poly-PR cytoplasmic aggregates can form by clustering around stress granules (SGs) and can nucleate cytoplasmic TDP-43 aggregates, while maintaining a TDP-43/DPR demixed state. These findings unveil a converging molecular mechanism for aberrant C9orf72-DPR and TDP-43 species—formation of ordered nuclear condensates. Furthermore, they link putative early disease-stage C9-ALS/FTD species to TDP-43 pathology—the main correlate of neurodegeneration. Finally, they provide a possible mechanism of large DPR inclusion nucleation/seeding in the cytoplasm.

## Results

### Optogenetic modeling of C9orf72 poly-PR condensation in cells

**Figure 1:**
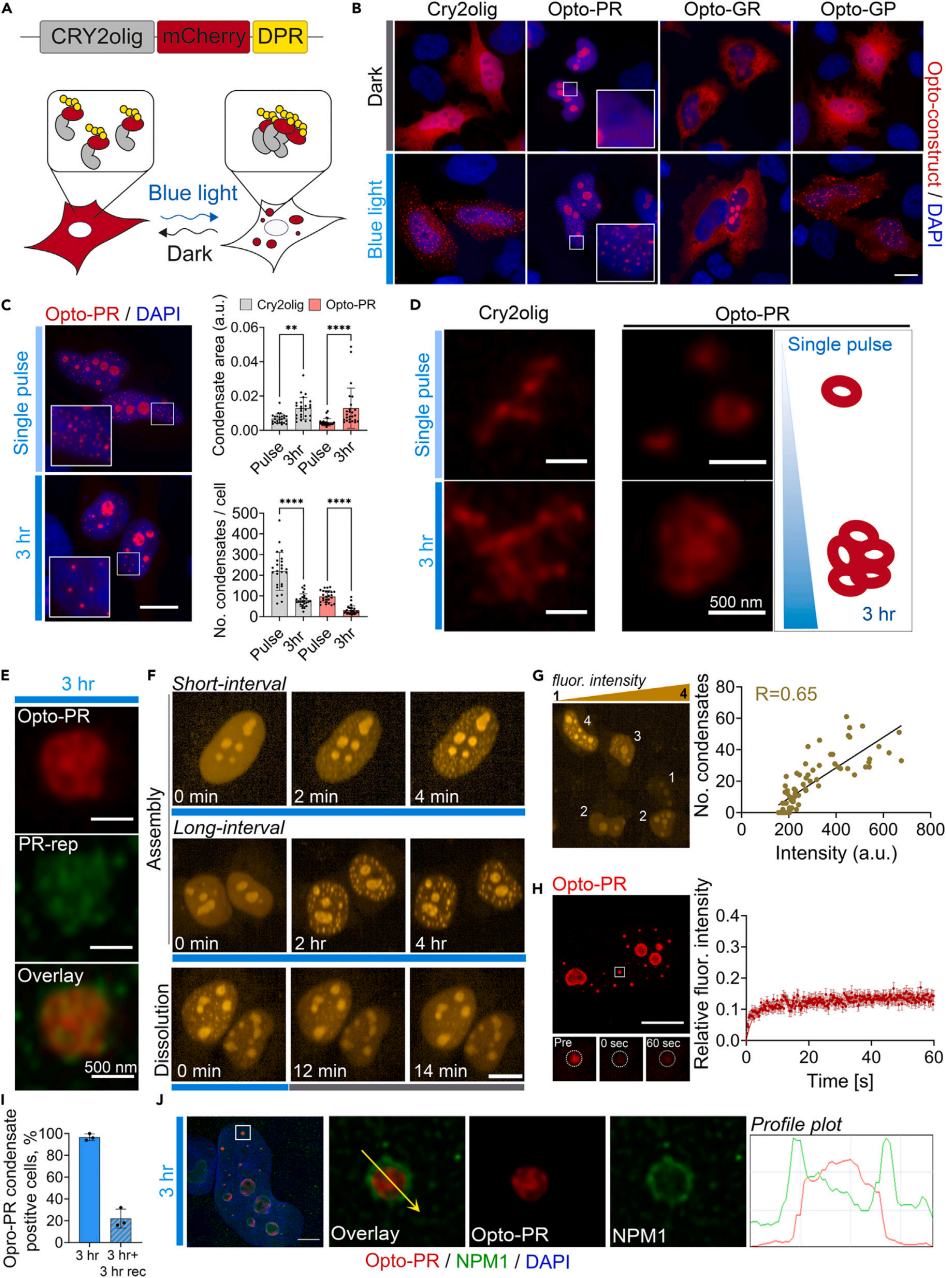
An optogenetic cellular system for controllable *C9orf72* DPR condensation (A) Opto-DPR approach. (B) Opto-DPR condensation can be induced by a single-pulse blue light stimulation. HeLa cells expressing the respective opto-DPR_(x36)_ or empty Cry2olig-mCherry vector were analyzed 24 h post-transfection. Blue-light array (single-pulse) was used. Scale bar, 10 μm. (C) Continuous opto-stimulation induces larger opto-PR condensates as compared to single light pulse. HeLa cells expressing opto-PR or empty vector were subjected to either a single-pulse or a 3-h continuous blue-light stimulation. 30 cells per condition were analyzed from a representative experiment. Error bars represent S.D. ∗∗*p* < 0.01, ∗∗∗∗*p* < 0.0001, Kruskal-Wallis test with Dunn’s post-hoc test. Scale bar, 10 μm. (D) Super-resolution microscopy (SRM) demonstrates structural differences between Cry2olig-only and opto-PR assemblies and between the condensates formed after single-pulse and continuous (3-h long) blue-light stimulation. Representative images and graphical representation are shown. (E) Opto-PR condensates can be detected using a PR-repeat specific antibody. Representative image (confocal single optical section) is shown. (F) Opto-PR condensate induction and tracking using confocal longitudinal imaging. Opto-PR expressing cells were stimulated with a 488 nm laser (at 80% for 500 ms), coupled with mCherry imaging. Cells were stimulated/imaged either every 2 min (“short-interval”) or every 15 min for up to 4 h (“long-interval”). Alternatively, cells with preformed condensates were imaged every 2 min without stimulation (“dissolution”). Representative images are shown. Scale bar, 10 μm. (G) Opto-PR condensate nucleation is concentration-dependent. mCherry fluorescence intensity was measured in the nucleoplasm of individual cells, outside the nucleolus, and the number of condensates was quantified in the same cells at the peak of their assembly (7-min interval repetitive stimulation for 49 min). 75 cells were analyzed. Also see Figure S1C. (H) FRAP analysis after full opto-PR condensate photobleaching reveals low protein mobility between the condensate and nucleoplasm. Representative image and FRAP curve for 25 cells from a representative experiment are shown. Error bars represent SEM. Scale bar, 10 μm. (I) Nuclear opto-PR condensates can become persistent. Opto-PR condensate were induced by 3-h continuous stimulation on blue-light array and the proportion of condensate-positive cells was quantified immediately post-stimulation or after 3 h of recovery in the dark. *N* = 3 (300 cells analyzed in total). Error bars represent S.D. (J) Opto-PR condensates are positive for nucleophosmin (NPM1). Opto-PR condensates induced by a 3-h continuous opto-stimulation were analyzed by SRM. Representative images and profile plots are shown. Scale bar, 2 μm.

We utilized Cry2olig, a Cry2 variant with high oligomerization capacity,^34^ in an attempt to induce and maintain R-DPR self-association. “Opto-DPR” constructs were generated for codon-optimized expression of poly-PR, poly-GR, and additionally poly-GP (36 repeats), tagged with Cry2olig and mCherry on the N-terminus (Figure 1A). Opto-DPRs demonstrated a subcellular distribution pattern in HeLa cells typical for the 30–1000 repeat range,^23^^,^^25^^,^^35^^,^^36^ where poly-GR and -GP were predominantly cytoplasmic and poly-PR was predominantly nuclear, with high enrichment in the nucleolus; none of the DPRs showed signs of aggregation (Figure 1B). Upon single-pulse light stimulation on a custom blue-light array, Cry2olig vector control, opto-PR and opto-GP but not opto-GR readily formed visible clusters/foci outside the nucleoli (Figure 1B), consistent with the low poly-GR self-association capacity reported in simulation studies.^26^ Opto-PR foci were small (<500 nm), dot-like and exclusively nuclear, whereas opto-GP formed large amorphous cytoplasmic inclusions (Figure 1B). We also tested Cry2PHR^37^ which is less oligomerisation-prone, as an opto-tag for poly-PR. However, Cry2PHR-tagged poly-PR displayed filamentous nuclear aggregates in a large fraction of cells even with no light stimulation (data not shown) and therefore could not be used for controllable self-assembly induction. We focused on opto-PR (poly-PR with Cry2olig tag) thereafter, using opto-GR as a non-aggregating control.

Nuclear opto-PR foci/condensates were negative for the two nucleolar markers tested, fibrillarin (FBL) and UPF1, confirming that they are not merely fragments of the nucleolus (Figure S1A). Continuous 3-h blue-light stimulation led to an increased opto-PR condensate size, as compared to single pulse (Figure 1C). By super-resolution microscopy (SRM), the condensates induced by a single light pulse were found to represent a mixed population of ∼100 nm dot-like and ∼250 nm spherical, hollow-center assemblies resembling anisotropic vesicle-like structures formed by arginine-rich peptide/RNA mixtures *in vitro.*^38^ Furthermore, the larger condensates formed after 3-h continuous light exposure was found to represent multiples of these spheroids (Figure 1D). Although Cry2olig protein on its own also formed clusters throughout the cell in response to blue light (Figure 1B),^34^ these structures appeared disordered/filamentous and were clearly different from opto-PR condensates (Figure 1D). Opto-PR condensates could be detected by a PR-repeat specific antibody used in human tissue studies (Figure 1E). Therefore, poly-PR confers a specific architecture to light-inducible condensates.

We next set up an imaging approach for simultaneous induction and tracking of opto-PR condensation on a confocal high-content imaging system. Even extremely short blue-light exposures and low 488 nm laser power (50 ms/5%) were sufficient to induce opto-PR foci; a combination of 500 ms exposure/80% laser power was used in subsequent experiments as consistently and robustly inducing condensates (Figure S1B). Visible opto-PR condensates appeared within 2 min post-pulse and could be maintained by both short- and long-interval repetitive opto-stimulation (every 2 min or every 15 min, respectively, Figure 1F). Condensates were reversible, typically resolving within ∼14 min after the last light pulse (Figure 1F). Opto-PR condensate nucleation was concentration-dependent, with more structures forming in higher-expressing cells (R = 0.65) (Figures 1G and S1C). Fluorescent recovery after photobleaching (FRAP) analysis revealed limited dynamics of opto-PR within these assemblies, with low recovery after photobleaching of the entire structure, despite a significant amount of diffuse opto-PR in the nucleoplasm (Figure 1H). A fraction of cells (22.0 ± 8.7%) developed persistent opto-PR condensates after continuous light stimulation, which were still detectable after 3 h of recovery in the dark (Figure 1I).

Poly-PR was previously shown to interact with nucleophosmin (NPM1) and to co-partition with this protein into phase-separated droplets *in vitro.*^29^^,^^39^ Consistent with this, opto-PR condensates stained positive for NPM1, where NPM1 formed a “shell” around the condensates, suggesting its secondary recruitment (Figure 1J). Interestingly, opto-stimulation induced opto-PR signal segregation in the nucleolus (Figure S1D). In this, we observed intra-nucleolar demixing of opto-PR from NPM1, where the proteins fully co-localized in the granular component (GC) under dark conditions but formed two distinct phases after opto-stimulation (Figures S1E and S1F). In contrast, opto-PR and FBL (the latter residing in the nucleolar dense fibrillar component, DFC) showed no co-localization both under dark and light conditions (Figures S1E and S1F).

R-DPRs were shown to promiscuously interact with membraneless organelle (MLO) components—RNA and proteins with low-complexity domains, leading to wide-spread MLO dysfunction.^12^^,^^23^^,^^29^^,^^40^ We investigated the effect of opto-PR and its condensation on MLOs, focusing on those in the nucleus due to the predominantly nuclear localization of this DPR. Systematic analysis of four MLOs—nuclear bodies (Gems, Cajal bodies, paraspeckles, and speckles) revealed no changes in their number/size the presence of diffuse or condensed opto-PR (Figure S2). Cytoplasmic SGs were not induced by opto-PR with or without blue-light stimulation (3 h continuous), consistent with its mainly nuclear localization (data not shown).

Thus, microscopically visible DPR self-assembly/condensation in cultured cells can be achieved using Cry2olig tagging, allowing the formation of DPR-specific, ordered assemblies. Opto-PR condensates are characterized by concentration-dependent growth and low dynamic properties associated with persistence, and sequester NPM1.

### RNA limits the growth of anisotropic poly-PR condensates in cells

**Figure 2:**
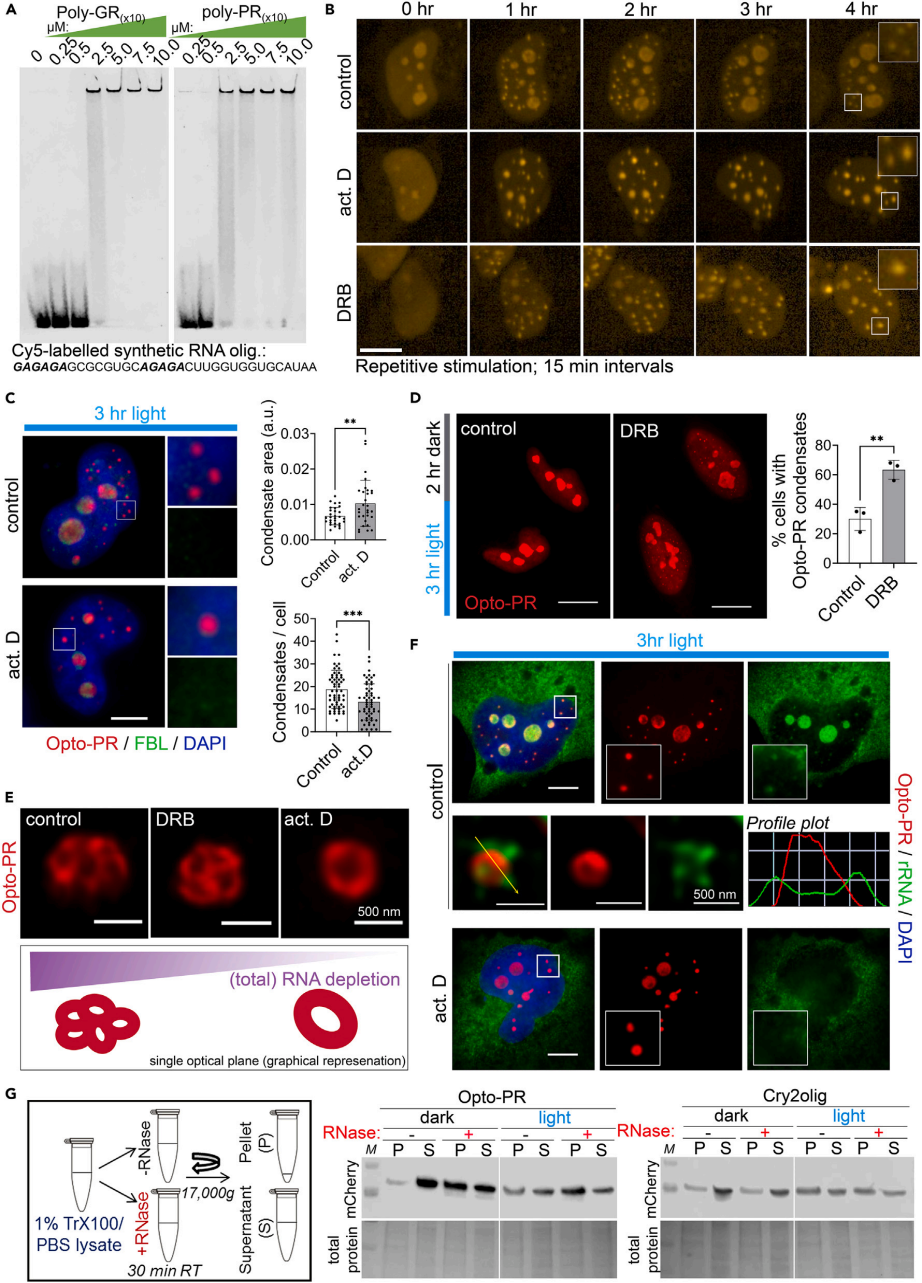
RNA limits opto-PR condensation in the nucleus (A) Electrophoretic mobility shift assay (EMSA) with a natural GA-rich RNA sequence reveals R-DPR binding to RNA. Cy5-labeled synthetic nucleotide “Clip34nt” (34-mer) and synthetic poly-PR and poly-GR peptides (10 repeats) were used. Representative gel is shown. (B) RNA depletion promotes opto-PR condensate growth. Opto-PR expressing cells were pre-treated with actinomycin D or DRB for 1 h, followed by long-interval repetitive blue-light stimulation (every 15 min) coupled with time-lapse imaging for up to 4 h (in the presence of the inhibitor). Representative images are shown. Scale bar, 10 μm. (C) Opto-PR condensates formed under conditions of actinomycin D-induced RNA depletion are larger in size and less numerous than in RNA-sufficient cells. Cells were opto-stimulated for 3 h continuously. Representative images and quantification are shown. Note that the larger condensates remain FBL-negative. 30 and 60 cells (5–7 fields) per condition were included in analysis for condensate size and number, respectively, from a representative experiment. Error bars represent S.D. ∗∗*p* < 0.01, ∗∗∗*p* < 0.001, Student’s t test. Scale bar, 5 μm. (D) Opto-PR condensates formed in RNA-depleted conditions are more persistent. Opto-PR expressing cells were light-stimulated for 3 h continuously, with or without DRB addition, followed by DRB removal and recovery for 2 h in the dark. Representative images and quantification are shown. *N* = 3 (50 cells from 5 to 7 fields of view analyzed per biological repeat). Error bars represent S.D. ∗∗*p* < 0.01, unpaired *t* test. Scale bar, 10 μm. (E) Opto-PR condensates formed in actinomycin D-treated cells are structurally different, as revealed by SRM. Representative images of condensates of a comparable size from control, DRB- or actinomycin D-treated cells induced by 3-h continuous opto-stimulation are shown, alongside with graphical representation. (F) Ribosomal (r)RNA depletion from the nucleus and opto-PR condensates in actinomycin D treated cells. Representative images are shown. Scale bar, 5 μm. (G) RNA degradation in the lysate promotes opto-PR insolubility. Lysates of opto-stimulated and control cells were subjected to RNase A digest for 30 min, with subsequent fractionation by centrifugation. S, supernatant; P, pellet.

We next asked whether our opto-model can be used to characterize potential modifiers of poly-PR condensation. RNA was previously found to promote R-DPR phase separation *in vitro.*^27^^,^^28^^,^^41^ More recently, using RNA-protein crosslinking, R-DPRs have been shown to bind RNA in cells, in particular ribosomal RNA (rRNA), with a preference for GA-rich sequences.^27^^,^^42^ Using electrophoretic mobility shift assay (EMSA) with an RNA oligonucleotide representing a naturally occurring RNA sequence containing 5xGA repeats (Clip34nt),^43^ we indeed observed that synthetic poly-PR and -GR peptides (10-mers) form complexes with RNA (Figure 2A).

Having confirmed RNA binding, we next studied the impact of RNA depletion on opto-PR condensates. Opto-PR expressing cells were treated with two transcriptional blockers, a global inhibitor (actinomycin D) and an RNA polymerase II-specific inhibitor (5,6-dichloro-1-β-D-ribofuranosylbenzimidazole, DRB), followed by induction and tracking of opto-PR condensates in individual cells for up to 4 h (long-interval repetitive stimulation, in the presence of the inhibitor). Both inhibitors caused dramatic nucleolar shrinking confirming their activity in cells (Figure 2B).^44^ Opto-PR condensates formed in RNA-depleted conditions appeared noticeably larger compared to control (Figure 2B). Similar result was obtained with a 3-h continuous opto-stimulation on blue-light array, and quantification confirmed a significant increase in the opto-PR condensate size (Figure 2C). These larger structures remained negative for the nucleolar marker FBL (Figure 2C). DRB is a reversible inhibitor, which allowed us to analyze possible changes in the stability of RNA-depleted opto-PR condensates. Cells were stimulated for 3 h in the presence or absence of DRB followed by 2 h of recovery in the dark. Opto-PR condensates formed in the presence of DRB appeared significantly more persisting, still detectable following the recovery in 63 ± 6.4% cells, compared to 30 ± 7.8% cells in control condition (Figure 2D).

Increase in the opto-PR condensate size upon actinomycin D treatment was accompanied by a decrease in their number (Figure 2C), suggesting clustering or fusion. SRM analysis revealed that opto-PR condensates in actinomycin D treated cells were no longer clusters of individual ∼250 nm spherical units seen in the untreated or DRB condition but instead represented larger (>500 nm) hollow-center spheres (Figure 2E). RNA on the surface of condensates has been shown to limit their fusion, including in cells.^45^ Actinomycin D but not DRB depletes ribosomal RNA (rRNA), and we observed dense rRNA signal on the opto-PR condensate surface which disappeared after actinomycin D treatment (Figure 2F). Therefore, the opto-PR condensates in actinomycin D treated cells may form by fusion of the smaller units due to rRNA depletion from the surface, followed by relaxation into a larger spherical assembly.

To further explore the role for RNA in poly-PR condensation, we used an orthogonal, biochemical approach. Lysates of cells expressing opto-PR or Cry2olig alone were subjected to RNase A digest, fractionated into soluble and pellet fractions and analyzed by western blot. Opto-PR significantly shifted to the pellet fraction after opto-stimulation, and RNA degradation promoted its insolubility under both dark and light conditions; in contrast, Cry2olig alone was not sensitive to RNase A digest (Figure 2G).

Another putative modifier of R-DPR self-association/condensation is arginine dimethylation (DMA), which increases the fluidity of R-DPR droplets *in vitro* and was found enriched in R-DPR inclusions in C9-ALS/FTD patient tissue.^41^ We employed two small molecule methyltransferase inhibitors, MS023 inhibiting five type-I methyltransferases that synthesize asymmetric DMA (aDMA) and EPZ015666, a specific inhibitor of PRMT5 responsible for most symmetric DMA (sDMA).^46^^,^^47^ Opto-PR expressing cells were treated with MS023 and EPZ015666 for 24 h and then exposed to blue-light for 3 h continuously, followed by opto-PR condensate quantification. We observed a mild decrease in the condensate number after aDMA depletion without changes in their size or structure (Figure S3). Thus, removing DMA marks may attenuate opto-PR condensate nucleation, although this effect is small.

Therefore, our cellular opto-PR model can be utilized to analyze modifiers of poly-PR self-assembly in the cellular context, as exemplified by the modulatory effect of RNA that we have uncovered.

### Poly-PR condensation induces nuclear TDP-43 pathology

**Figure 3:**
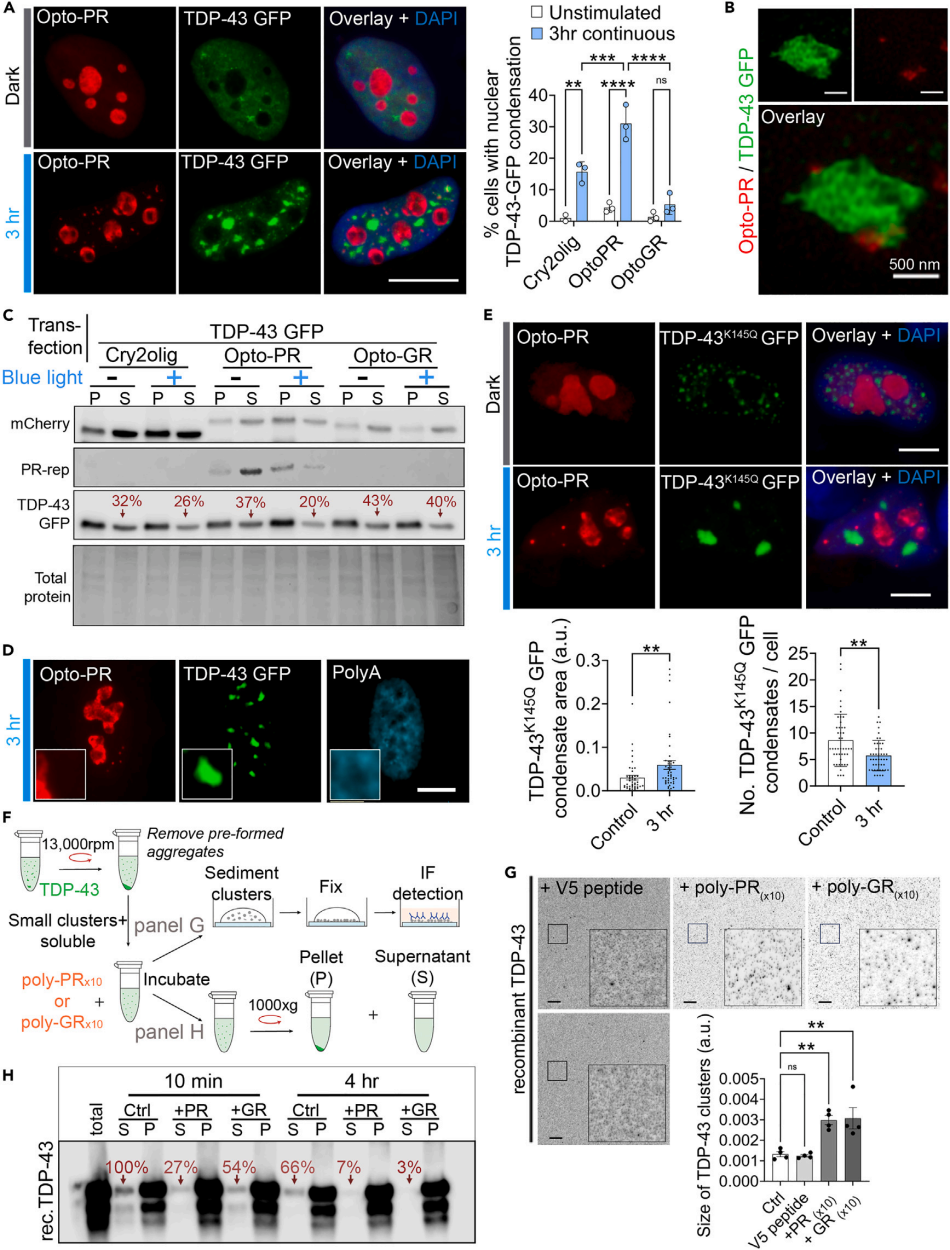
Nuclear opto-PR condensation induces TDP-43 pathology (A) Opto-PR self-assembly induces nuclear condensation of co-expressed TDP-43 GFP. Cells were opto-stimulated for 3 h continuously. Representative images and quantification are shown. Scale bar, 10 μm. *N* = 3 (≥100 cells from 5 to 7 fields of view analyzed per biological repeat). Error bars represent S.D. ∗∗*p* < 0.01, ∗∗∗*p* < 0.001, ∗∗∗∗*p* < 0.0001, mixed-effects model analysis with Tukey’s multiple comparisons test. (B) Spatial proximity of opto-PR and TDP-43 GFP nuclear condensates as revealed by SRM. Representative image is shown. (C) Reduced TDP-43 GFP solubility upon induction of opto-PR condensation. Cells co-expressing opto-PR and TDP-43 GFP were subjected to 3-h continuous blue-light stimulation before lysis and fractionation by centrifugation. Western blots and quantification of the relative TDP-43 GFP amount in the supernatant fraction are shown for a representative experiment. S, supernatant; P, pellet. (D) TDP-43 condensates induced by opto-PR self-assembly are devoid of polyA + RNA. Cells after 3-h continuous opto-stimulation were analyzed by poly(dT) RNA-FISH. Representative image is shown. Scale bar, 5 μm. (E) Opto-PR self-assembly promotes nuclear condensation of a TDP-43 acetylation mimic mutant (K145Q). Representative images and quantification are shown. 50 cells (5–7 fields of view) were analyzed from a representative experiment. Error bars represent S.D. ∗∗*p* < 0.01, Mann-Whitney *U* test. Scale bar, 5 μm. (F–H) R-DPRs promote TDP-43 clustering *in vitro.* Recombinant TDP-43 (supernatant fraction depleted of large aggregates; 1 μM) was incubated with equimolar amounts of poly-PR_(x10)_ or poly-GR_(x10)_ peptides or control peptide (V5) and analyzed by immunostaining and fractionation/western blot. Methodology (F), immunostaining/imaging (G) and fractionation/western blotting (H) data are shown. In (G), 10 min incubation was used and 4 fields of view from a representative experiment were analyzed; ∗∗*p* < 0.01, one-way ANOVA with Dunnett’s post-hoc test. Error bars represent S.D. Bottom left image is a control without any peptide added. In (H), representative western blot and band intensity quantification for the supernatant are shown. S, supernatant; P, pellet. Scale bar, 10 μm.

R-DPR interactomes are enriched in RNA-binding proteins (RBPs).^12^^,^^29^ We therefore examined the effect of nuclear opto-PR condensation on ALS/FTD relevant RBPs. TDP-43, FUS, NONO, and SFPQ, all tagged with GFP, were co-expressed with opto-PR and their subcellular distribution was examined with and without opto-stimulation. Opto-PR presence *per se* did not affect RBP distribution (Figures 3A and S4A). However, light-stimulated opto-PR expressing cells displayed a striking nuclear condensation phenotype for TDP-43 but no other RBPs analyzed (Figures 3A and S4A). Although we did observe TDP-43 condensation in a fraction of light-stimulated Cry2olig-expressing, it was significantly smaller than in opto-PR cultures; nor was it observed in light-stimulated opto-GR expressing cells (Figure 3A). Opto-PR assemblies were frequently found in the physical contact with TDP-43 condensates (44 ± 0.5% of all opto-PR foci) (Figure 3B), suggestive of a direct nucleating effect of oligomerizing opto-PR. Fractionation confirmed reduced solubility of TDP-43 GFP after induction of opto-PR condensation but not in light-stimulated opto-GR expressing cells (Figure 3C).

TDP-43 GFP nuclear condensates induced by opto-PR were similar in their morphology to the condensates formed during the recovery from arsenite stress.^48^^,^^49^^,^^50^ Like these stress-induced foci, TDP-43 condensates induced by opto-PR were devoid of polyA + RNA (Figure 3D). TDP-43 acetylation was shown to impair its RNA binding and enhance aggregation, with acetylated TDP-43 inclusions detected in sALS.^49^ We investigated the effect of opto-PR condensation on an acetylation-mimic, TDP-43 mutant K145Q. In agreement with the published data, TDP-43 K145Q was prone to spontaneously forming nuclear foci/granules in naive cells (Figure S4D). Opto-PR condensation potentiated the pro-aggregating effect of this mutation, further increasing its nuclear granulation (Figure 3E). In co-immunoprecipitation experiments, we also found that TDP-43 pull-down by opto-PR was more efficient under the conditions of RNA depletion (Figure S4E). In contrast, and consistent with intact FUS distribution in cells with opto-PR condensates (Figure S4A), FUS was undetectable in opto-PR pull-downs (Figure S4E).

We next examined whether TDP-43 condensation elicited by opto-PR was due to an upregulated stress response. Classic cellular stress markers, phosho-eIF2α, GADD34, and ATF4, were not altered in opto-PR expressing cells after 3-h light stimulation (Figures S4B and S4C). This is in contrast to the dramatic upregulation of these markers during the recovery from sodium arsenite stress used as a positive control (Figure S4C). In addition to demonstrating that opto-PR self-assembly directly induces TDP-43 granulation, this experiment also confirmed a lack of phototoxicity in our model.

Finally, we investigated the effect of R-DPRs on TDP-43 higher-order assembly *in vitro*, using the condensate immunodetection/imaging assay we recently developed^50^^,^^51^ (Figure 3F). Both poly-PR and poly-GR peptides were included in these studies. “Supernatant” fraction of recombinant TDP-43 (after removal of preformed aggregates) containing small protein clusters and soluble protein was incubated with equimolar amounts of synthetic R-DPR peptides, followed by sedimentation and fixation of TDP-43 clusters on coverslips for immunostaining/imaging; in parallel, samples were fractionated by centrifugation for western blot analysis (Figure 3F). As a control for molecular crowding, we used a peptide with a “generic” sequence moderately enriched in proline and containing no arginine residues (V5: GKPIPNPLLGLDST). Addition of both R-DPRs significantly enhanced TDP-43 clustering—manifested as an increased cluster size (Figure 3G). Fractionation confirmed increased partitioning of TDP-43 to the pellet fraction in the presence of R-DPRs (Figure 3H).

Collectively, these results suggest that poly-PR condensation can directly cause changes to the nuclear distribution of TDP-43 without activation of stress signaling, where RNA depletion promotes poly-PR interaction with TDP-43.

### Cytoplasmic poly-PR assemblies are persistent and selectively sequester TDP-43

**Figure 4:**
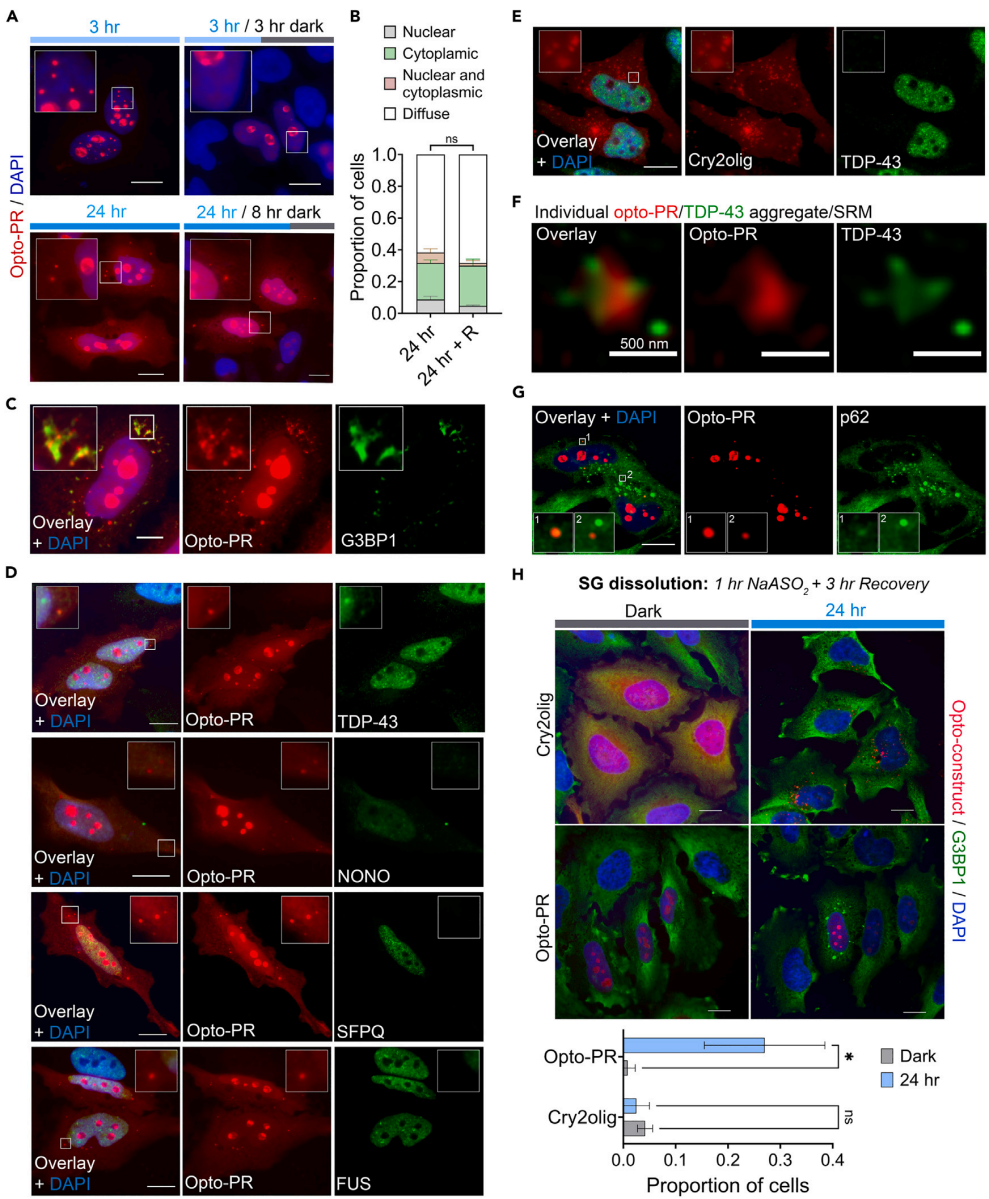
Prolonged opto-stimulation elicits cytoplasmic opto-PR/TDP-43 pathology (A and B) Prolonged, 24-h blue-light stimulation leads to cytoplasmic opto-PR redistribution and aggregation, with persistent assembly formation. Proportion of cells with opto-PR assemblies (nuclear and cytoplasmic) was quantified after 24-h blue-light array stimulation with and without 8-h recovery in the dark. Representative images (A) and quantification (B) are shown. Images for a 3-h stimulation and recovery are included for comparison. *N* = 3 (300 cells analyzed per condition). Error bars represent S.D. ns, non-significant. Scale bar, 10 μm. (C) Cytoplasmic opto-DPR foci surround spontaneous stress granules (SGs, visualized using G3BP1 as a marker). Representative image is shown. Cells were opto-stimulated for 24 h continuously. Scale bar, 5 μm. (D) Endogenous TDP-43 but, no other RBPs, joins opto-PR assemblies induced by prolonged opto-stimulation. Also note normal nuclear localization of all RBPs in cells with cytoplasmic opto-PR. Cells were opto-stimulated for 24 h continuously. Representative images are shown. Scale bars, 10 μm. (E) Cry2olig-only cytoplasmic assemblies are negative for TDP-43. Cells were opto-stimulated for 24 h continuously. Representative images are shown. Scale bars, 10 μm. (F) TDP-43 remains demixed within opto-PR within cytoplasmic assemblies, as revealed by SRM. Cells were opto-stimulated for 24 h continuously. Representative image is shown. (G) Cytoplasmic opto-PR assemblies are occasionally positive for p62. Representative image is shown. Insets 1 and 2 shows examples of p62-positive and -negative assemblies, respectively. Scale bars, 10 μm. (H) SG dissolution is affected in cells with cytoplasmically localized opto-PR. SGs Representative were induced NaAsO_2_ in cells expressing either opto-PR or Cry2olig-only, subjected to 24-h long opto-stimulation or kept in the dark. Efficiency of SG dissolution was analyzed 3 h into the recovery (post-NaAsO_2_ removal). Representative images and quantification are shown. *N* = 3 (120 cells analyzed per condition). Error bars represent S.D. ∗*p* < 0.05, two-way ANOVA with Sidak’s multiple comparisons test. Scale bars, 10 μm.

Having characterized the nuclear phenotypes, we asked whether our opto-PR model is amenable to reproducing the cytoplasmic pathology typical for DPRs. Continuous 24-h long stimulation on the blue-light array resulted in significant redistribution of opto-PR to the cytoplasm, with cytoplasmic foci formation in 32% of cells (>300 transfected cells analyzed; Figures 4A and 4B). This was accompanied by a reduction in the incidence of nuclear opto-PR condensates (from 94% cells after 3 h to 9% after 24 h of stimulation) (Figures 4A, 4B, and 1I). Nuclear and cytoplasmic opto-PR foci induced by 24-h opto-stimulation were persistent, with no significant decline observed after 8 h of recovery in the dark (Figures 4A and 4B). This is in contrast to the nuclear opto-PR condensates forming after a 3-h stimulation that were largely cleared after 3 h of recovery in the dark (Figures 4A and 1I). Furthermore, we found that in a small proportion of cells that developed spontaneous SGs, opto-PR assemblies surrounded SGs (Figure 4C). Opto-PR redistribution to the cytoplasm was not due to the nuclear membrane damage/nuclear pore complex disruption, since nuclear retention of several RBPs was not affected in these cells (Figure 4D). Therefore, impaired nuclear import of opto-PR under these conditions could be due to its submicroscopic oligomerization in the cytoplasm. Strikingly, endogenous TDP-43 but no other ALS-related RBPs (FUS, NONO, and SFPQ) were found to be enriched in the cytoplasmic opto-PR foci (Figure 4D). In contrast, Cry2olig-only cytoplasmic structures induced by 24-h light stimulation were negative for TDP-43 (Figure 4E). SRM revealed that cytoplasmic opto-PR assemblies were ∼250 nm structures that, unlike nuclear condensates, were not hollow (Figure 4F). It also revealed that in these assemblies, opto-PR and TDP-43 remained demixed, with the TDP-43 signal primarily on the surface (Figure 4F). A small fraction of cytoplasmic opto-PR foci were positive for p62 (Figure 4G) but none of them stained positive for ubiquitin (data not shown).

We next examined whether opto-PR accumulated/aggregated in the cytoplasm after prolonged opto-stimulation affects stress-induced SGs.^28^^,^^52^^,^^53^ Opto-PR and Cry2olig-only expressing cells subjected to long opto-stimulation or kept in the dark were analyzed during the recovery from NaAsO_2_ (3 h time point), using G3BP1 as a marker. SG disassembly was delayed in light-stimulated opto-PR expressing cells compared to unstimulated opto-PR cells or Cry2-only expressing (stimulated or unstimulated) cells (Figure 4H). This finding further validates our cellular model as capable of reproducing the key molecular effects of R-DPRs on the cellular RNA/RNP granule metabolism.

Therefore, our opto-model is amenable to the induction of cytoplasmic poly-PR accumulation and aggregation, with its cytoplasmic assemblies being significantly more persistent than those in the nucleus. Our data also point to a role for SGs in the growth of cytoplasmic poly-PR aggregates as well as a role for poly-PR assemblies in “nucleating” TDP-43 aggregation in the cytoplasm.

### Poly-PR condensation in cultured human motor neurons

**Figure 5:**
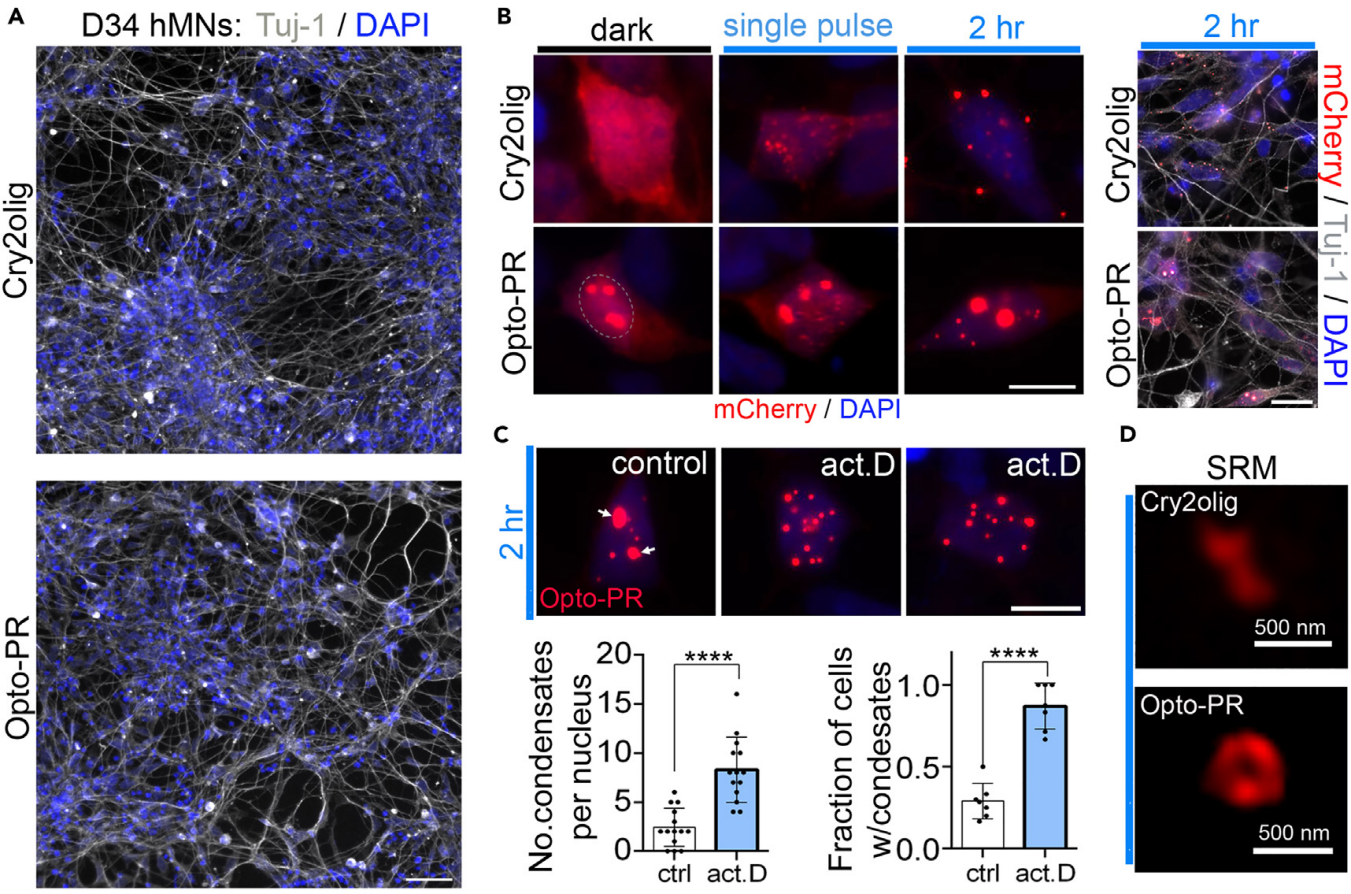
Opto-PR condensation in human motor neurons (hMNs) (A) Day 34 hMN cultures transduced with Cry2olig or opto-PR. Neuronal cultures stained for Tuj-1 imaged 48 h post-transduction demonstrate similar cell density. Scale bar, 50 μm. (B) Distribution and light-induced condensation of lentivirus-delivered Cry2olig and opto-PR expression constructs in hMNs. Note significant accumulation of opto-PR in the cytoplasm in the steady-state. 48 h post-transduction, neurons were either subjected to single pulse of blue-light and fixed after 4 min, or stimulated with blue-light for 2 h. General plane images of stimulated cultures co-stained for Tuj-1 are also shown (right). Dashed line indicates the nucleus. Scale bar, 5 μm (left) and 15 μm (right). (C) RNA depletion promotes opto-PR condensation in hMNs. Neurons were pre-treated with actinomycin D for 2, before a 2-h long blue-light stimulation. Number of condensates/nucleus was quantified for 15 neurons per condition. Arrows indicate nucleoli in untreated cells (note that due to nucleoli shrinking, they are indistinguishable by size from large opto-PR condensates). Fraction of cells was quantified from 7 fields of view (42 and 34 cells for control and actinomycin (D), respectively). Error bars represent S.D. ∗∗∗∗*p* < 0.0001, two-way *t* test. Scale bar, 5 μm. (D) Opto-PR condensate morphology in hMNs, as revealed by SRM analysis. Neurons were subjected to blue-light stimulation for 2 h. Representative images are shown.

In order to verify the reproducibility of the previous phenotypes in a disease-relevant cell type, we employed lentiviral delivery of opto-PR or Cry2olig-only expression constructs. Postmitotic human motor neurons differentiated from a stem cell line and characterized previously^54^ (Figure 5A) were transduced at day 34 and analyzed 48 h post-transduction. Although the distribution of Cry2olig alone in neurons was similar to HeLa cells, opto-PR displayed more prominent cytoplasmic localization (Figure 5B). Due to higher sensitivity to phototoxicity in neurons, as determined in preliminary experiments, neurons were stimulated on blue-light array for a maximum of 2 h. Both single-pulse and 2-h continuous blue-light stimulation induced opto-PR condensation in neurons, primarily in the nucleus, with condensates growing in size during prolonged stimulation (Figure 5B). RNA depletion by actinomycin D treatment significantly potentiated opto-PR condensation, where not only the size but also number of nuclear condensates significantly increased after actinomycin D pre-treatment (Figure 5C). Finally, using SRM, we confirmed that opto-PR condensates in neurons also possess the hollow-center structure (Figure 5D). Thus, the core features of light-inducible opto-PR condensation are reproducible in human motor neurons.

## Discussion

A vast body of knowledge on C9-DPR related disease mechanisms has accumulated in the past decade yet it remains unclear whether, and if so how, DPR self-assembly, resulting in a C9-ALS/FTD hallmark pathology, contributes to the disease. Aggregation intermediates, and especially smaller, highly reactive oligomeric species, have been validated as toxic/pathogenic in the case of other neurodegeneration-linked proteins, such as tau and alpha-synuclein.^55^^,^^56^^,^^57^ It is possible that equivalent DPR aggregation products play a role in C9-ALS/FTD. Poly-PR is the least abundant DPR in human tissue^8^^,^^58^ despite theoretically it should be expressed at the levels similar to other DPRs (except poly-GP produced from both strands). Although this can be due to a variation in the expression mechanisms and antibody detection, it is also possible that neurons that accumulate poly-PR are lost early in disease due to its high toxicity, including its aggregation products. Although the inclusions of all five DPRs in the patient CNS are morphologically similar,^7^ DPRs other than GA fail to form microscopically visible assemblies in cell models.^21^^,^^23^^,^^59^ In order to circumvent the high solubility of R-DPRs in cells, we harnessed the Cry2olig opto-module^34^ successfully used previously to promote self-association of neurodegeneration-linked proteins.^60^^,^^61^^,^^62^ In line with the molecular dynamics predictions,^26^ poly-GR’s self-association capacity was too low even when facilitated by Cry2olig, not yielding visible condensation in cells. However, we succeeded in achieving the condensation of the oligomerisation-competent poly-PR, including in cultured human motor neurons.

Two key phenotypes—alterations to the nucleolus and SGs—validate our opto-PR model in the context of the existing literature.^12^^,^^39^^,^^52^ In our model, SG disassembly was impaired by cytoplasmic opto-PR (but not Cry2olig-only). This can be caused by altered composition, and hence dynamics, of SGs formed in the opto-PR rich milieu. We also observed sequestration of NPM1, a confirmed R-DPR interactor, into nuclear opto-PR condensates. Beyond these two phenotypes, however, we failed to detect the ubiquitous MLO disruption by poly-PR reported in some studies, and its condensation also had limited effect on MLOs. This may be due to high variability of these phenotypes depending on the cell type, DPR levels and repeat length. It would be important to establish consensus phenotypes that can be used for the benchmarking of novel cellular DPR models, and the nucleolar and SG pathology validated in multiple studies are the prime candidate readouts.

We found that poly-PR confers a specific ordered arrangement to the opto-induced nuclear condensates—sphere with a hollow center. Arginine-rich peptides form such anisotropic structures in the presence of RNA *in vitro*, both under RNA and peptide excess conditions.^38^ Adopting this model, we speculate that upon interaction with cellular RNA, the small opto-PR oligomers nucleated with the aid of Cry2olig, form nanocondensates with a neutral “head” and a charged “tail” that subsequently coalesce into RNA-coated micelles (∼100 nm granules). These transitions into a vesicle-like conformation “layered” with RNA on both the internal and external surface (∼250 nm granules). Upon (r)RNA depletion, these condensates undergo fusion into a larger (>500 nm) hollow-center structure (Figure S5A). Importantly, these phenotypes are recapitulated in cultured human motor neurons. Poly-PR was shown to form nuclear structures in C9-ALS/FTD, varying from compact inclusions to less dense “territories”, in multiple studies.^5^^,^^53^^,^^58^^,^^63^ Nuclear poly-PR condensates not overlapping with nucleolar markers also form in transgenic mice.^64^ It would be interesting to establish whether the structures seen in mice adopt a hollow-center structure during their biogenesis. Anisotropic nuclear assemblies (“anisosomes”) are formed by acetylated TDP-43 in cultured cells and *in vivo.*^33^ RBPs complexed with polyadenylated RNA form similar structures in cell models of spinal muscular atrophy.^65^ Furthermore, nuclear condensates of RNA-binding deficient TDP-43 and ALS-linked CREST protein^66^ also possess this typical morphology (Figure S5B). Remarkably, DDX3X mutations causative of neurodevelopmental disorders also form cytoplasmic hollow condensates, and those composed of an aggressive RNA-binding deficient mutant display low recovery in FRAP.^67^ It is possible that specific changes to the cellular metabolism in neurological disease, e.g., altered protein and RNA stoichiometries, favor this assembly type. RNA facilitates LLPS of poly-PR *in vitro*,^27^ however, cellular RNA restricts the growth of the non-dynamic opto-PR condensates in cells. Together with the reports on the solubilizing effect of RNA on RBPs^62^^,^^68^^,^^69^^,^^70^ and on the wide-spread RNA degradation in ALS,^71^ our findings suggest than declining RNA levels may be a common factor underlying protein aggregation across ALS subtypes including C9-ALS.

Transient nuclear TDP-43 condensation is a hallmark of stress response.^48^ We recently found that this molecular event leads to TDP-43 loss of function and prolonged STMN2 depletion and is dysregulated by TDP-43 mutations.^50^ These phenotypes may become persistent with chronic or repetitive stress, precipitating the disease. Indeed, recent use of a specific RNA aptamer revealed abnormal nuclear TDP-43 granulation in motor neurons in ALS tissue.^72^ Here, we show that poly-PR self-assembly can cause TDP-43 condensation in the absence of stress signaling. *In vitro*, poly-PR and -GR promote TDP-43 clustering (this study) and reduce its solubility^73^ equally well. This suggests that in cells, (competing) interactions with other proteins will modulate the effect of the two R-DPRs.

Cytoplasmic poly-PR assemblies in our opto-model are structurally dissimilar to and more persistent than the nuclear condensates, probably reflecting a different environment in the two cellular compartments. This higher stability is consistent with a higher frequency of poly-PR cytoplasmic inclusions in patients.^6^ DPR inclusions are rarely found to co-deposit with TDP-43 inclusions in the patient tissue, with clear anatomical region specificity.^58^^,^^74^ Some histopathological studies found evidence of DPR aggregation preceding the cytoplasmic TDP-43 pathology.^75^^,^^76^ TDP-43 joins the cytoplasmic opto-PR foci where the two proteins remained in two phases. It is tempting to speculate that transient cytoplasmic poly-PR assemblies “seed” TDP-43 pathology. SGs may play an equivalent role for cytoplasmic poly-PR inclusions, concentrating the initial poly-PR assemblies in a confined space and promoting their coalescence into a larger aggregate. Indeed, a recent study showed that cytoplasmic TDP-43 aggregates can be nucleated within SGs, as a separate phase, and left behind after SG dissolution.^77^ Inclusions in C9-ALS/FTD are likely composed of several DPRs, and their co-expression leads to different phenotypes *in vivo.*^78^ Opto-GP readily undergoes cytoplasmic condensation in our model, enabling future opto-DPR co-aggregation studies.

### Limitations of the study

We used a relatively short repeat length (which nevertheless is in the patient range), whereas the Cry2olig-mCherry is a large tag. However, multiple studies have demonstrated that DPR repeat lengths of 30–1000 have identical subcellular localization with similar toxicity.^12^^,^^35^^,^^39^^,^^79^ We cannot exclude that longer repeats will undergo condensation more readily, which should be tested in future studies. In addition, for this proof-of-principle study, non-neuronal cells were used in the majority of experiments for the ease of expression and imaging. Further characterization of this system is neuronal models and *in vivo* is warranted.

## Resource availability

### Lead contact

Requests for reagents and further information should be directed to the Lead Contact, Tatyana A. Shelkovnikova (t.shelkovnikova@sheffield.ac.uk).

### Materials availability

All study materials are available from the corresponding authors; a signed Material Transfer Agreement (MTA) may be required for transfer.

### Data and code availability

• Data reported in the paper will be shared by the lead contact upon request.
• This paper does not report original code.
• Any additional information required to reanalyze the data reported in this work paper is available from the lead contact upon request.

## Acknowledgments

This work was supported by the UKRI Future Leaders Fellowship (MR/W004615/1), 10.13039/501100000265MRC (MR/W028522/1) and 10.13039/501100000268BBSRC (BB/V014110/1) standard grants and MND Association fellowship/grant (968-799) to T.A.S. and 10.13039/501100000268BBSRC (BB/S005277/1) grant to G.M.H. B.C.S.E. is funded by an MND Scotland PhD studentship. We also acknowledge the 10.13039/501100000265MRC grant MR/X012077/1 for Airyscan 2 LMF.

## Author contributions

R.E.H: conceptualization; data curation; formal analysis; investigation; methodology; writing—original draft; writing—review and editing. J.A.R.: formal analysis; investigation; methodology. W.H.: formal analysis; investigation; methodology. A.S.A.: formal analysis; investigation. B.C.S.E.: investigation. Y.L.: investigation. N.S.: investigation. G.M.H.: supervision; funding acquisition. T.A.S.: conceptualization; supervision; funding acquisition; project administration; writing—review and editing.

## Declaration of interests

Authors declare no competing interests.

## STAR★Methods

### Key resources table

**Table:** 

REAGENT or RESOURCE	SOURCE	IDENTIFIER
**Antibodies**		

FBL (rabbit polyclonal)	Proteintech	Cat# 16021-1-AP; RRID: AB_2105788
NPM1 (mouse monoclonal)	Proteintech	Cat# 60096-1-Ig; RRID: AB_2155162
UPF1 (rabbit polyclonal)	Proteintech	Cat# 23379-1-AP; RRID: AB_11232421
PNN (rabbit polyclonal)	Proteintech	Cat# 18266-1-AP;RRID: AB_10642138
coilin p80 (mouse monoclonal)	BD Biosciences	Cat# 612074; RRID: AB_2081554
SMN (mouse monoclonal)	BD Biosciences	Cat# 610647; RRID: AB_397973
G3BP1 (rabbit polyclonal)	Proteintech	Cat# 13057-2-AP; RRID: AB_2232034
repeat-PR (rabbit polyclonal)	Proteintech	Cat# 23979-1-AP; RRID: AB_2879388
TDP-43 (rabbit polyclonal)	Sigma	Cat# T1580; RRID: AB_2532125
TDP-43 (mouse monoclonal)	R&D Biosystems	Cat# MAB77781
FUS (mouse monoclonal)	Santa Cruz	Cat# sc-47711; RRID: AB_2105208
NONO (rabbit polyclonal)	Proteintech	Cat# 11058-1-AP; RRID: AB_2152167
SFPQ (rabbit monoclonal)	Abcam	Cat# ab177149; RRID: AB_2910265
Ribosomal RNA (mouse monoclonal)	Bio-Techne	Cat# NB100-662; RRID: AB_10000550
p62/SQSTM1 (mouse monoclonal)	R&D Biosystems	Cat# MAB8028R; RRID: AB_2885151
mCherry (rabbit polyclonal)	Proteintech	Cat# 26765-1-AP; RRID: AB_2876881
GFP (mouse monoclonal)	Proteintech	Cat# 66002-1-Ig; RRID: AB_11182611
eIF2α (rabbit polyclonal)	Cell Signaling	Cat# 9722; RRID: AB_2230924
eIF2αP (rabbit polyclonal)	Abcam	Cat# ab32157; RRID: AB_732117
Beta III Tubulin/Tuj-1 (chicken)	Merck	Cat# AB9354; RRID: AB_570918
Secondary fluorescently labeled antibodies: Alexa 488/546 Fluor anti-mouse/rabbit IgG	ThermoFisher	Cat# A-11008; A-11001; A-11030; A-11035; RRID: AB_143165, RRID: AB_2534069, RRID: AB_2737024, RRID: AB_143051
Mouse IgG HRP Linked Whole Ab	Amersham	Cat# NA931; RRID: AB_772210
Rabbit IgG HRP Linked Whole Ab	Amersham	Cat# NA934; RRID: AB_772206

**Bacterial and virus strains**		

NEB® Stable Competent E. coli (High Efficiency)	New England Biolabs	Cat# C3040H
NEB® 5-alpha Competent E. coli (High Efficiency)	New England Biolabs	Cat# C2987H

**Chemicals, peptides, and recombinant proteins**		

Actinomycin D	Sigma-Aldrich	Cat# A1410
5,6-dichloro-1-beta-D-ribofuranosylbenzimidazole	Sigma-Aldrich	Cat# D1916
MS-023	ApexBio	Cat# B7448
EPZ015666	ApexBio	Cat# B4989
NaAsO_2_	Sigma-Aldrich	Cat# S7400
Bovine Serum Albumin (BSA)	Jackson ImmunoResearch	Cat# 001-000-161
RNase A	New England Biolabs	Cat#T3018L
Recombinant TDP-43	R&D Biosystems	Cat# AP-190-100
Poly-PR_x10_ peptide (custom-made)	BioSynth	N/A
Poly-PR_x10_ peptide (custom-made)	BioSynth	N/A
V5 peptide GKPIPNPLLGLDST	Proteintech	Cat# v5p
Poly-L-lysine	Sigma-Aldrich	Cat# P4832
Aqueous Glutaraldehyde EM Grade	EMS Diasum	Cat#16110

**Critical commercial assays**		

Lipofectamine2000 Transfection Reagent	ThermoFisher	Cat# 11668027
M-MLV Reverse Transcriptase	Promega	Cat# M1701
qPCRBIO SyGreen Mix Lo-Rox	PCR Biosystems	Cat# PB20.11–05
JetPRIME Transfection Reagent	PolyPlus	Cat# 101000027
GenElute™ Mammalian Total RNA Miniprep Kit	Sigma-Aldrich	Cat# RTN350
QIAzol Lysis Reagent	Qiagen	Cat# 79306
Chromotek RFP-Trap agarose	Proteintech	Cat# rta
10% Mini-PROTEAN® TGX™ Precast Gel, 15-well	Bio-Rad	Cat# 4561036
Clarity Max Western ECL Substrate	Bio-Rad	Cat# 1705062
GelCode™ Blue Safe Protein Stain	ThermoFisher	Cat# 24594
Epredia™ Immu-Mount™	ThermoFisher	Cat# 10622689
Matrigel matrix	Corning	Cat# 356234
B27 supplement	ThermoFisher	Cat# 12587010
BDNF	Stemcell Technologies	Cat# # 78005

**Experimental models: Cell lines**		

Human: HeLa cells (ATCC)	Sigma-Aldrich	Cat#93021013
Human: motor neurons (Day 34) derived from ES H9 line	Shelkovnikova et al.^54^	N/A

**Oligonucleotides**		

Oligo(dT)30-Cy5 DNA FISH probe	Sigma	N/A
Random hexamers	ThermoFisher	Cat# 48190011
Primers for qRT-PCR	Shelkovnikova et al.^54^	N/A
Clip34nt RNA oligonucleotide: [CY5] (GAGAGAGCGCGUGC AGAGACUUGGUGGUGCAU AA) [BIOTEG]	Eurofins (custom-synthesised). This paper	N/A

**Recombinant DNA**		

Plasmid: CRY2olig-mCherry (pmCherryN1)	Taslimi et al.^34^ (Addgene plasmid)	Cat# 60032
Plasmid: pCRY2PHR-mCherryN1 (pmCherryN1)	Kennedy et al.^37^ (Addgene plasmid)	Cat# 26866
Plasmid: CRY2olig-mCherry (pmCherryN1) with PR_x36_ insert	This paper	N/A
Plasmid: pCRY2PHR-mCherryN1-PR_x36_	This paper (available at Addgene)	Cat# 218917
Plasmid: pCRY2PHR-mCherryN1-GP_x36_	This paper (available at Addgene)	Cat# 218918
Plasmid: pCRY2PHR-mCherryN1-GR_x36_	This paper (available at Addgene)	Cat# 218919
Plasmid: CRY2olig-mCherry in lentiviral vector (pLvos)	This paper	N/A
Plasmid: CRY2olig-mCherry-PR_x36_ in lentiviral vector (PLvos)	This paper	N/A
Plasmid: TDP-43 WT GFP (pEGFP-C1)	Kukharsky et al.^66^	N/A
Plasmid: FUS-GFP (pEGFP-C1)	Shelkovnikova et al.^80^	N/A
Plasmid: NONO-GFP (pEGFP-C1)	Kukharsky et al.^66^	N/A
Plasmid: SFPQ-GFP (pEGFP-C1)	Lee et al.^81^	N/A
Plasmid: CREST-GFP (pEGFP-C1)	Kukharsky et al.^66^	N/A
Plasmid: TDP-43 K145Q GFP (pEGFP-C1)	This paper	N/A
Plasmid: TDP-43 F417/149L GFP (pEGFP-C1)	This paper	N/A

**Software and algorithms**		

Harmony 4.9 High-Content Imaging and Analysis Software	PerkinElmer	https://www.revvity.com/gb-en/category/cellular-imaging-software
ZEN blue software	ZEISS	https://www.zeiss.com/microscopy/en/products/software/zeiss-zen.html
GraphPad Prism 9	GraphPad Software, Inc	https://www.graphpad.com/updates/prism-900-release-notes
CellSens Dimension software	Olympus	https://www.olympus-lifescience.com/en/software/cellsens/
ImageJ	National Institutes of Health	https://imagej.net/ij/

**Other**		

Olympus BX57 upright microscope equipped with ORCA-Flash 4.0 camera (Hamamatsu)	Olympus (Evident)	Custom
ZEISS LSM 800 confocal microscope with Airyscan 2	Zeiss	https://www.zeiss.com/microscopy/en/products/light-microscopes/confocal-microscopes.html
PerkinElmer Opera Phenix - HCS	PerkinElmer	https://www.revvity.com/gb-en/product/opera-phenix-plus-system-hh14001000
CFX96 Touch Real-Time PCR Detection System	Bio-Rad	https://www.bio-rad.com/ru-ru/product/cfx96-touch-real-time-pcr-detection-system?ID=LJB1YU15
PhenoPlate-96, black, optically clear bottom	PerkinElmer	Cat# 6055302

### Experimental model and study participant details

HeLa cells were obtained from ATCC via Sigma, cultured in Dulbecco’s Modified Eagle Medium/Nutrient Mixture F-12 (DMEM/F-12) supplemented with 10% foetal bovine serum (FBS) and penicillin-streptomycin. Human motor neurons were differentiated from H9 hES cell line as described.^54^

### Method details

#### Plasmids

The CRY2olig-mCherry backbone vector was from Addgene (plasmid #60032).^34^ Plasmids with codon-optimized DPR_x36_ sequences were a gift from Kurt De Vos.^82^ DPR sequences were cloned into the CRY2olig-mCherry vector using standard techniques and sequences were verified by Sanger sequencing. These plasmids are available via Addgene (#218917, 218918, 218918). Plasmids for the expression of GFP-tagged TDP-43, FUS, NONO, and CREST (N-terminal tag, in pEGFP-C1 vector) were generated previously.^66^^,^^80^ SFPQ-GFP plasmid is a gift from Archa Fox.^81^ TDP-43 K145Q and F147/149L in pEGFP-C1 vector were generated using PCR mutagenesis.

#### Cell culture, transfection and treatments

For time-lapse imaging, cells were plated on PhenoPlate-96 (black, optically clear bottom, PerkinElmer) at a density of 2 × 10^4^. For all other experiments, cells were seeded in 24 well plates, with or without coverslips dependent on the application, at a density of 5 × 10^4^ cells, unless otherwise stated. Transfection was performed 24 h prior to blue light stimulation, using either Lipofectamine 2000 (ThermoFisher) or jetPRIME (Jena Bioscience) according to the manufacturer’s instructions. For transcriptional inhibition cells were treated with 2.5 μg/mL actinomycin D or 5,6-dichloro-1-beta-D-ribofuranosylbenzimidazole (DRB) (both Sigma). For inhibition of arginine methylation, cells were treated with 10 μM MS-023 or EPZ015666 (both ApexBio). To induce cellular stress, cells were treated with 500 μM NaAsO_2_ (Sigma). Incubation times for treatments within individual experiments are indicated in respective Figure legends.

#### Opto-stimulation

Cells expressing opto-constructs were stimulated with a 488 nm laser on Opera Phenix HCS (500 ms, 80% laser power) for live cell time-lapse imaging (under full environmental control conditions), or on a custom-built LED blue-light array housed in a humidified incubator maintained at 37°C with 5% CO_2_. Cells were protected from light between experiments and during fixation.

#### Generation of lentiviral constructs and neuronal transduction

Cry2olig-mCherry and Cry2olig-mCherry-PR_x36_ cassettes were subcloned into pLvos lentiviral vector^83^ (gift from Kurt De Vos) using standard cloning techniques. For lentivirus production, 20 × 10 cm dishes of HEK293T cells (3 × 10^6^ per dish) cells/dish were each transfected with 13 μg pCMVΔR8.92, 3.75 μg pMDG, 3 μg pRSV and 13 μg pLvos-Cry2olig-mCherry or pLvos-Cry2olig-mCherry-PR_x36_ plasmids using calcium phosphate transfection. Media was replaced after 16 h. After a further 48 h, the supernatant was collected, filtered through a 0.45 μm filter and centrifuged at 19,000 rpm for 90 min at 4°C. The viral pellet was re-suspended in 1xPBS with 1% BSA and stored at −80°C. The biological titer was determined by qPCR using WPRE primers to estimate the integration level of lentiviral DNA from SH-SY5Y cells transduced with a range of lentivirus dilutions and subsequent microscopy analysis of mCherry fluorescence. Human motor neurons were seeded onto Matrigel-coated coverslips at a density of 90 × 10^3^ at day 23, matured for 10 days and transduced with either opto-PR and control vector at MOI 5. Medium was changed the following day, and the cultures were opto-stimulated and analyzed 48 h post-transduction. Due to higher phototoxicity than HeLa cells, the maximum opto-stimulation time for neurons was 2 h.

#### Immunocytochemistry and RNA-FISH

Immunocytochemistry was performed as described elsewhere^54^^,^^84^ using the following commercially available antibodies: FBL (rabbit polyclonal, Proteintech, 16021-1-AP); NPM1 (mouse monoclonal, Proteintech, 60096-1-Ig); UPF1 (rabbit polyclonal, Proteintech, 23379-1-AP); PNN (rabbit polyclonal, Proteintech, 18266-1-AP); coilin p80 (mouse monoclonal, BD Biosciences, 612074); SMN (mouse monoclonal, BD Biosciences, 610647); G3BP1 (rabbit polyclonal, Proteintech, 13057-2-AP); repeat-PR (rabbit polyclonal, Proteintech, 23979-1-AP); TDP-43 (rabbit polyclonal C-terminal, Sigma); FUS (mouse monoclonal, Santa Cruz, [4H11], sc-47711); NONO (rabbit polyclonal, Proteintech, 11058-1-AP); SFPQ (rabbit monoclonal, Abcam, ab177149); ribosomal RNA (mouse monoclonal Y10b, NB100-662); p62/SQSTM1 (mouse monoclonal, MAB8028R, R&D Biosystems); Tuj-1 (chicken, AB9354, Merck). RNA-FISH using a Cy5-labelled dT_(30)_ DNA oligonucleotide probe (Sigma) was performed as described earlier.^54^

#### Microscopy

Conventional fluorescence microscopy was performed using 100× objective on an Olympus BX57 upright microscope equipped with an ORCA-Flash 4.0 camera (Hamamatsu) and cellSens Dimension software (Olympus). Super-resolution microscopy was performed using a 63× oil immersion objective on a ZEISS 980 laser scanning confocal microscope (LSM) with Airyscan 2 detector and ZEN Blue software. Time-lapse microscopy was performed using a 40× objective on Opera Phenix HCS, and Harmony 4.9 software was used for image processing and analysis (all PerkinElmer). Image processing and profile drawing was done using ImageJ or ZEN Blue software. Condensate/aggregate quantification was done manually or on ImageJ in a blinded manner.

#### Fluorescent recovery after photobleaching (FRAP)

Cells seeded at a density of 2.8 × 10^5^ in glass-bottomed 35 mm dishes (Ibidi), were transfected and 24 h post-transfection, subjected to 3-h continuous stimulation prior to FRAP analysis. Imaging was performed using a 63× oil immersion objective on a ZEISS LSM 800 confocal microscope, equipped with a humidified incubation chamber maintained at 37°C with 5% CO_2_. FRAP acquisition was performed on condensates formed after 3 h of continuous stimulation. A circular region of interest (ROI) around each condensate was bleached using a 568 nm laser at 100% laser power. Images were captured pre-bleach, immediately following bleach and at ∼200 ms intervals during recovery. The mean fluorescence intensity within the ROI was determined for each image using ZEN blue software. Intensity values were corrected for bleaching during imaging and normalized to pre-bleach intensity. Average values were plotted and FRAP curves fitted using a one-phase association equation in GraphPad Prism 9 software.

#### Electrophoretic mobility shift assay (EMSA)

Cy5-labeled RNA oligonucleotide 5′- GAGAGAGCGCGUGCAGAGACUUGGUGGUGCAUAA-3’ (“Clip34nt”) ^43^ was custom-synthesized by Eurofins. Poly-GR_x10_ and poly-PR_x10_ were custom-made by Biosynth. Lyophilized peptides were resuspended in water at 50 mM and stored at −80°C before use. RNA was incubated at 250 nM with 250 nM-10 μM peptides in the EMSA buffer (50 mM Tris-HCl, pH 7.5, 100 mM KCl, 2 mM MgCl_2_, 100 mM β-mercaptoethanol, 0.1 mg/mL BSA) for 15 min at RT with gentle shaking. Samples were analyzed on 6% native acrylamide gel in TBE buffer, followed by imaging on Licor Odissey FC (700 nm channel).

#### Cell lysis, fractionation, co-immunoprecipitation and western blotting

For fractionation, cells were lyzed in 1% Triton X-100 in 1xPBS by incubating on ice for 30 min with vortexing at 5 min intervals, followed by centrifuging at 13,000 rpm for 20 min at 4°C. For RNase A treatment, lysates were split in half and RNase A (10 μg/mL) was added to one-half, with both tubes incubated at RT for 30 min. Supernatant and pellet were mixed with 2× Laemmli buffer, vortexed and heated at 95°C for 10 min. For co-IP, the lysates were cleared by at 13,000 rpm for 20 min at 4°C and the supernatants were incubated with RFP-Trap beads (Chromotek), in low-binding tubes, on a nutator, for 3 h. Beads were washed in lysis buffer 3 times and protein was eluted in 2× Laemmli buffer. For western blotting, proteins were resolved on a 10% Mini-PROTEAN TGX hand-cast protein gel and transferred to a PVDF membrane. Gels were stained with Gelcode (ThermoFisher) post-transfer for total protein. Membranes were blocked for 1 h in 4% milk/TBST and then incubated with the following primary antibodies (1:1000 dilution) at 4°C overnight: mCherry (rabbit polyclonal, Proteintech, 26765-1-AP); GFP (mouse monoclonal, 66002-1-Ig, Proteintech); TDP-43 (rabbit polyclonal, C-terminal, Sigma); eIF2α (rabbit polyclonal, Cell Signaling, 9722), eIF2αP (rabbit polyclonal, Abcam, antibody [E90], ab32157); repeat-PR (rabbit polyclonal, Proteintech, 23979-1-AP). Following primary antibody incubation, blots were washed with TBST and incubated with an appropriate HRP secondary antibody (GE Healthcare) for 1 h at room temperature. Signal was detected using Clarity Max Western ECL Substrate (Bio-Rad) and imaged and quantified using Licor Odissey FC/Image Studio software.

#### RNA expression analysis

Total RNA was extracted using GenElute total mammalian RNA kit (Sigma) in accordance with the manufacturer’s instructions. First-strand cDNA synthesis was performed using 500 ng of RNA with random primers (ThermoFisher) and MMLV reverse transcriptase (Promega). qRT-PCR was performed using qPCRBIO SyGreen Lo-ROX (PCRbio), and GAPDH was used for normalization. Primer sequences are provided in a previous study.^54^

#### *In vitro* analysis of TDP-43 condensation

*In vitro* TDP-43 clustering analysis with immunodetection and imaging were performed as described in Huang et al and Hodgson et al.^50^^,^^51^ Briefly, 1 μM of recombinant TDP-43 (R&D Biosystems, AP-190-100; “soluble” fraction – supernatant after centrifuging at 13,300 rpm for 1 min) was mixed with 1 μM poly-PR/GR peptides (as above, BioSynth) or a generic peptide (V5: GKPIPNPLLGLDST, Proteintech) in the assay buffer, and incubated for 10 min. Samples were sedimented and fixed with glutaraldehyde on coverslips, blocked with 1% BSA in PBS for 1 h at RT and incubated with an anti-TDP-43 antibody (1:5000, mouse monoclonal, R&D Biosystems, MAB77781) in the blocking buffer for 2 h. After washes, TDP-43 protein clusters were visualized using anti-mouse Alexa Fluor 488 antibody (1:2000, ThermoFisher), incubated for 1 h at RT. Coverslips were mounted on a glass slide using Immu-mount (ThermoFisher). Images were taken using Olympus BX57 upright microscope and ORCA-Flash 4.0 camera and processed using cellSens Dimension software (Olympus). Quantification of assemblies was done using the ‘Analyze particles’ tool of ImageJ. For confirmatory western blot analysis, recombinant TDP-43 samples incubated with or without the peptides as above (for 10 min, and an additional set – for 4 h) were centrifuged at 1,000xg for 10 min. Pellet and supernatant were analyzed by western blot using a C-terminal TDP-43 antibody (Sigma).

### Quantification and statistical analysis

Analysis was done using respective tests on GraphPad Prism 9 software. N corresponds to the number of biological replicates and number of technical replicates (e.g., fields of view) is indicated in figure legends. Error bars represent standard deviation (S.D.) unless indicated otherwise. Details of statistical tests for each figure are provided in Table S1.

## Supplemental information

Document S1. Figures S1–S5

Table S1. Details of statistical tests, related to Figures 1–5 and Figures S2–S4

## References

[1] DeJesus-Hernandez M., Mackenzie I.R., Boeve B.F., Boxer A.L., Baker M., Rutherford N.J., Nicholson A.M., Finch N.A., Flynn H., Adamson J. (2011). Expanded GGGGCC hexanucleotide repeat in noncoding region of C9ORF72 causes chromosome 9p-linked FTD and ALS. Neuron.

[2] Renton A.E., Majounie E., Waite A., Simón-Sánchez J., Rollinson S., Gibbs J.R., Schymick J.C., Laaksovirta H., van Swieten J.C., Myllykangas L. (2011). A hexanucleotide repeat expansion in C9ORF72 is the cause of chromosome 9p21-linked ALS-FTD. Neuron.

[3] Van Mossevelde S., van der Zee J., Cruts M., Van Broeckhoven C. (2017). Relationship between C9orf72 repeat size and clinical phenotype. Curr. Opin. Genet. Dev..

[4] Ash P.E.A., Bieniek K.F., Gendron T.F., Caulfield T., Lin W.L., Dejesus-Hernandez M., van Blitterswijk M.M., Jansen-West K., Paul J.W., Rademakers R. (2013). Unconventional translation of C9ORF72 GGGGCC expansion generates insoluble polypeptides specific to c9FTD/ALS. Neuron.

[5] Mori K., Weng S.M., Arzberger T., May S., Rentzsch K., Kremmer E., Schmid B., Kretzschmar H.A., Cruts M., Van Broeckhoven C. (2013). The C9orf72 GGGGCC repeat is translated into aggregating dipeptide-repeat proteins in FTLD/ALS. Science.

[6] Zu T., Liu Y., Bañez-Coronel M., Reid T., Pletnikova O., Lewis J., Miller T.M., Harms M.B., Falchook A.E., Subramony S.H. (2013). RAN proteins and RNA foci from antisense transcripts in C9ORF72 ALS and frontotemporal dementia. Proc. Natl. Acad. Sci. USA.

[7] Gendron T.F., Bieniek K.F., Zhang Y.J., Jansen-West K., Ash P.E.A., Caulfield T., Daughrity L., Dunmore J.H., Castanedes-Casey M., Chew J. (2013). Antisense transcripts of the expanded C9ORF72 hexanucleotide repeat form nuclear RNA foci and undergo repeat-associated non-ATG translation in c9FTD/ALS. Acta Neuropathol..

[8] Mackenzie I.R.A., Frick P., Grässer F.A., Gendron T.F., Petrucelli L., Cashman N.R., Edbauer D., Kremmer E., Prudlo J., Troost D., Neumann M. (2015). Quantitative analysis and clinico-pathological correlations of different dipeptide repeat protein pathologies in C9ORF72 mutation carriers. Acta Neuropathol..

[9] Saberi S., Stauffer J.E., Jiang J., Garcia S.D., Taylor A.E., Schulte D., Ohkubo T., Schloffman C.L., Maldonado M., Baughn M. (2018). Sense-encoded poly-GR dipeptide repeat proteins correlate to neurodegeneration and uniquely co-localize with TDP-43 in dendrites of repeat-expanded C9orf72 amyotrophic lateral sclerosis. Acta Neuropathol..

[10] Sakae N., Bieniek K.F., Zhang Y.J., Ross K., Gendron T.F., Murray M.E., Rademakers R., Petrucelli L., Dickson D.W. (2018). Poly-GR dipeptide repeat polymers correlate with neurodegeneration and Clinicopathological subtypes in C9ORF72-related brain disease. Acta Neuropathol. Commun..

[11] Schludi M.H., May S., Grässer F.A., Rentzsch K., Kremmer E., Küpper C., Klopstock T., Arzberger T., Edbauer D., German Consortium for Frontotemporal Lobar Degeneration, Bavarian Brain Banking Alliance (2015). Distribution of dipeptide repeat proteins in cellular models and C9orf72 mutation cases suggests link to transcriptional silencing. Acta Neuropathol..

[12] Kwon I., Xiang S., Kato M., Wu L., Theodoropoulos P., Wang T., Kim J., Yun J., Xie Y., McKnight S.L. (2014). Poly-dipeptides encoded by the C9orf72 repeats bind nucleoli, impede RNA biogenesis, and kill cells. Science.

[13] Mizielinska S., Grönke S., Niccoli T., Ridler C.E., Clayton E.L., Devoy A., Moens T., Norona F.E., Woollacott I.O.C., Pietrzyk J. (2014). C9orf72 repeat expansions cause neurodegeneration in Drosophila through arginine-rich proteins. Science.

[14] Moens T.G., Niccoli T., Wilson K.M., Atilano M.L., Birsa N., Gittings L.M., Holbling B.V., Dyson M.C., Thoeng A., Neeves J. (2019). C9orf72 arginine-rich dipeptide proteins interact with ribosomal proteins in vivo to induce a toxic translational arrest that is rescued by eIF1A. Acta Neuropathol..

[15] Xu L., Wang D., Zhao L., Yang Z., Liu X., Li X., Yuan T., Wang Y., Huang T., Bian N. (2023). C9orf72 poly(PR) aggregation in nucleus induces ALS/FTD-related neurodegeneration in cynomolgus monkeys. Neurobiol. Dis..

[16] LaClair K.D., Zhou Q., Michaelsen M., Wefers B., Brill M.S., Janjic A., Rathkolb B., Farny D., Cygan M., de Angelis M.H. (2020). Congenic expression of poly-GA but not poly-PR in mice triggers selective neuron loss and interferon responses found in C9orf72 ALS. Acta Neuropathol..

[17] Zhang Y.J., Guo L., Gonzales P.K., Gendron T.F., Wu Y., Jansen-West K., O'Raw A.D., Pickles S.R., Prudencio M., Carlomagno Y. (2019). Heterochromatin anomalies and double-stranded RNA accumulation underlie C9orf72 poly(PR) toxicity. Science.

[18] Chew J., Cook C., Gendron T.F., Jansen-West K., Del Rosso G., Daughrity L.M., Castanedes-Casey M., Kurti A., Stankowski J.N., Disney M.D. (2019). Aberrant deposition of stress granule-resident proteins linked to C9orf72-associated TDP-43 proteinopathy. Mol. Neurodegener..

[19] Choi S.Y., Lopez-Gonzalez R., Krishnan G., Phillips H.L., Li A.N., Seeley W.W., Yao W.D., Almeida S., Gao F.B. (2019). C9ORF72-ALS/FTD-associated poly(GR) binds Atp5a1 and compromises mitochondrial function in vivo. Nat. Neurosci..

[20] Cook C.N., Wu Y., Odeh H.M., Gendron T.F., Jansen-West K., Del Rosso G., Yue M., Jiang P., Gomes E., Tong J. (2020). C9orf72 poly(GR) aggregation induces TDP-43 proteinopathy. Sci. Transl. Med..

[21] Frottin F., Perez-Berlanga M., Hartl F.U., Hipp M.S. (2021). Multiple pathways of toxicity induced by C9orf72 dipeptide repeat aggregates and G(4)C(2) RNA in a cellular model. Elife.

[22] Hartmann H., Hornburg D., Czuppa M., Bader J., Michaelsen M., Farny D., Arzberger T., Mann M., Meissner F., Edbauer D. (2018). Proteomics and C9orf72 neuropathology identify ribosomes as poly-GR/PR interactors driving toxicity. Life Sci. Alliance.

[23] Liu F., Morderer D., Wren M.C., Vettleson-Trutza S.A., Wang Y., Rabichow B.E., Salemi M.R., Phinney B.S., Oskarsson B., Dickson D.W., Rossoll W. (2022). Proximity proteomics of C9orf72 dipeptide repeat proteins identifies molecular chaperones as modifiers of poly-GA aggregation. Acta Neuropathol. Commun..

[24] Lopez-Gonzalez R., Lu Y., Gendron T.F., Karydas A., Tran H., Yang D., Petrucelli L., Miller B.L., Almeida S., Gao F.B. (2016). Poly(GR) in C9ORF72-Related ALS/FTD Compromises Mitochondrial Function and Increases Oxidative Stress and DNA Damage in iPSC-Derived Motor Neurons. Neuron.

[25] Vanneste J., Vercruysse T., Boeynaems S., Sicart A., Van Damme P., Daelemans D., Van Den Bosch L. (2019). C9orf72-generated poly-GR and poly-PR do not directly interfere with nucleocytoplasmic transport. Sci. Rep..

[26] Zheng S., Sahimi A., Shing K.S., Sahimi M. (2021). Molecular Dynamics Study of Structure, Folding, and Aggregation of Poly-PR and Poly-GR Proteins. Biophys. J..

[27] Balendra R., Ruiz de Los Mozos I., Odeh H.M., Glaria I., Milioto C., Wilson K.M., Ule A.M., Hallegger M., Masino L., Martin S. (2023). Transcriptome-wide RNA binding analysis of C9orf72 poly(PR) dipeptides. Life Sci. Alliance.

[28] Boeynaems S., Bogaert E., Kovacs D., Konijnenberg A., Timmerman E., Volkov A., Guharoy M., De Decker M., Jaspers T., Ryan V.H. (2017). Phase Separation of C9orf72 Dipeptide Repeats Perturbs Stress Granule Dynamics. Mol. Cell.

[29] Lee K.H., Zhang P., Kim H.J., Mitrea D.M., Sarkar M., Freibaum B.D., Cika J., Coughlin M., Messing J., Molliex A. (2016). C9orf72 Dipeptide Repeats Impair the Assembly, Dynamics, and Function of Membrane-Less Organelles. Cell.

[30] Quaegebeur A., Glaria I., Lashley T., Isaacs A.M. (2020). Soluble and insoluble dipeptide repeat protein measurements in C9orf72-frontotemporal dementia brains show regional differential solubility and correlation of poly-GR with clinical severity. Acta Neuropathol. Commun..

[31] Shin Y., Berry J., Pannucci N., Haataja M.P., Toettcher J.E., Brangwynne C.P. (2017). Spatiotemporal Control of Intracellular Phase Transitions Using Light-Activated optoDroplets. Cell.

[32] Park H., Kim N.Y., Lee S., Kim N., Kim J., Heo W.D. (2017). Optogenetic protein clustering through fluorescent protein tagging and extension of CRY2. Nat. Commun..

[33] Yu H., Lu S., Gasior K., Singh D., Vazquez-Sanchez S., Tapia O., Toprani D., Beccari M.S., Yates J.R., Da Cruz S. (2021). HSP70 chaperones RNA-free TDP-43 into anisotropic intranuclear liquid spherical shells. Science.

[34] Taslimi A., Vrana J.D., Chen D., Borinskaya S., Mayer B.J., Kennedy M.J., Tucker C.L. (2014). An optimized optogenetic clustering tool for probing protein interaction and function. Nat. Commun..

[35] Bennion Callister J., Ryan S., Sim J., Rollinson S., Pickering-Brown S.M. (2016). Modelling C9orf72 dipeptide repeat proteins of a physiologically relevant size. Hum. Mol. Genet..

[36] Kanekura K., Harada Y., Fujimoto M., Yagi T., Hayamizu Y., Nagaoka K., Kuroda M. (2018). Characterization of membrane penetration and cytotoxicity of C9orf72-encoding arginine-rich dipeptides. Sci. Rep..

[37] Kennedy M.J., Hughes R.M., Peteya L.A., Schwartz J.W., Ehlers M.D., Tucker C.L. (2010). Rapid blue-light-mediated induction of protein interactions in living cells. Nat. Methods.

[38] Alshareedah I., Moosa M.M., Raju M., Potoyan D.A., Banerjee P.R. (2020). Phase transition of RNA-protein complexes into ordered hollow condensates. Proc. Natl. Acad. Sci. USA.

[39] White M.R., Mitrea D.M., Zhang P., Stanley C.B., Cassidy D.E., Nourse A., Phillips A.H., Tolbert M., Taylor J.P., Kriwacki R.W. (2019). C9orf72 Poly(PR) Dipeptide Repeats Disturb Biomolecular Phase Separation and Disrupt Nucleolar Function. Mol. Cell.

[40] Lin Y., Mori E., Kato M., Xiang S., Wu L., Kwon I., McKnight S.L. (2016). Toxic PR Poly-Dipeptides Encoded by the C9orf72 Repeat Expansion Target LC Domain Polymers. Cell.

[41] Gittings L.M., Boeynaems S., Lightwood D., Clargo A., Topia S., Nakayama L., Troakes C., Mann D.M.A., Gitler A.D., Lashley T., Isaacs A.M. (2020). Symmetric dimethylation of poly-GR correlates with disease duration in C9orf72 FTLD and ALS and reduces poly-GR phase separation and toxicity. Acta Neuropathol..

[42] Ortega J.A., Sasselli I.R., Boccitto M., Fleming A.C., Fortuna T.R., Li Y., Sato K., Clemons T.D., Mckenna E.D., Nguyen T.P. (2023). CLIP-Seq analysis enables the design of protective ribosomal RNA bait oligonucleotides against C9ORF72 ALS/FTD poly-GR pathophysiology. Sci. Adv..

[43] Bhardwaj A., Myers M.P., Buratti E., Baralle F.E. (2013). Characterizing TDP-43 interaction with its RNA targets. Nucleic Acids Res..

[44] Shav-Tal Y., Blechman J., Darzacq X., Montagna C., Dye B.T., Patton J.G., Singer R.H., Zipori D. (2005). Dynamic sorting of nuclear components into distinct nucleolar caps during transcriptional inhibition. Mol. Biol. Cell.

[45] Cochard A., Garcia-Jove Navarro M., Piroska L., Kashida S., Kress M., Weil D., Gueroui Z. (2022). RNA at the surface of phase-separated condensates impacts their size and number. Biophys. J..

[46] Chan-Penebre E., Kuplast K.G., Majer C.R., Boriack-Sjodin P.A., Wigle T.J., Johnston L.D., Rioux N., Munchhof M.J., Jin L., Jacques S.L. (2015). A selective inhibitor of PRMT5 with in vivo and in vitro potency in MCL models. Nat. Chem. Biol..

[47] Eram M.S., Shen Y., Szewczyk M., Wu H., Senisterra G., Li F., Butler K.V., Kaniskan H.Ü., Speed B.A., Dela Seña C. (2016). A Potent, Selective, and Cell-Active Inhibitor of Human Type I Protein Arginine Methyltransferases. ACS Chem. Biol..

[48] Wang C., Duan Y., Duan G., Wang Q., Zhang K., Deng X., Qian B., Gu J., Ma Z., Zhang S. (2020). Stress Induces Dynamic, Cytotoxicity-Antagonizing TDP-43 Nuclear Bodies via Paraspeckle LncRNA NEAT1-Mediated Liquid-Liquid Phase Separation. Mol. Cell.

[49] Cohen T.J., Hwang A.W., Restrepo C.R., Yuan C.X., Trojanowski J.Q., Lee V.M.Y. (2015). An acetylation switch controls TDP-43 function and aggregation propensity. Nat. Commun..

[50] Huang W.P., Ellis B.C.S., Hodgson R.E., Sanchez Avila A., Kumar V., Rayment J., Moll T., Shelkovnikova T.A. (2024). Stress-induced TDP-43 nuclear condensation causes splicing loss of function and STMN2 depletion. Cell Rep..

[51] Hodgson R., Huang W.P., Kumar V., Haiyan A., Chalakova Z., Rayment J., Ellis B.C.S., Stender E.G.P., van Vugt J.J.F.A., Project MinE ALS Sequencing Consortium (2024). TDP-43 is a Master Regulator of Paraspeckle Condensation. SSRN.

[52] Marmor-Kollet H., Siany A., Kedersha N., Knafo N., Rivkin N., Danino Y.M., Moens T.G., Olender T., Sheban D., Cohen N. (2020). Spatiotemporal Proteomic Analysis of Stress Granule Disassembly Using APEX Reveals Regulation by SUMOylation and Links to ALS Pathogenesis. Mol. Cell.

[53] Wen X., Tan W., Westergard T., Krishnamurthy K., Markandaiah S.S., Shi Y., Lin S., Shneider N.A., Monaghan J., Pandey U.B. (2014). Antisense proline-arginine RAN dipeptides linked to C9ORF72-ALS/FTD form toxic nuclear aggregates that initiate in vitro and in vivo neuronal death. Neuron.

[54] Shelkovnikova T.A., Dimasi P., Kukharsky M.S., An H., Quintiero A., Schirmer C., Buée L., Galas M.C., Buchman V.L. (2017). Chronically stressed or stress-preconditioned neurons fail to maintain stress granule assembly. Cell Death Dis..

[55] Bengoa-Vergniory N., Roberts R.F., Wade-Martins R., Alegre-Abarrategui J. (2017). Alpha-synuclein oligomers: a new hope. Acta Neuropathol..

[56] Choi M.L., Gandhi S. (2018). Crucial role of protein oligomerization in the pathogenesis of Alzheimer's and Parkinson's diseases. FEBS J..

[57] Jucker M., Walker L.C. (2018). Propagation and spread of pathogenic protein assemblies in neurodegenerative diseases. Nat. Neurosci..

[58] Davidson Y., Robinson A.C., Liu X., Wu D., Troakes C., Rollinson S., Masuda-Suzukake M., Suzuki G., Nonaka T., Shi J. (2016). Neurodegeneration in frontotemporal lobar degeneration and motor neurone disease associated with expansions in C9orf72 is linked to TDP-43 pathology and not associated with aggregated forms of dipeptide repeat proteins. Neuropathol. Appl. Neurobiol..

[59] Zhou Q., Lehmer C., Michaelsen M., Mori K., Alterauge D., Baumjohann D., Schludi M.H., Greiling J., Farny D., Flatley A. (2017). Antibodies inhibit transmission and aggregation of C9orf72 poly-GA dipeptide repeat proteins. EMBO Mol. Med..

[60] Berard M., Sheta R., Malvaut S., Rodriguez-Aller R., Teixeira M., Idi W., Turmel R., Alpaugh M., Dubois M., Dahmene M. (2022). A light-inducible protein clustering system for in vivo analysis of alpha-synuclein aggregation in Parkinson disease. PLoS Biol..

[61] Jiang L., Lin W., Zhang C., Ash P.E.A., Verma M., Kwan J., van Vliet E., Yang Z., Cruz A.L., Boudeau S. (2021). Interaction of tau with HNRNPA2B1 and N(6)-methyladenosine RNA mediates the progression of tauopathy. Mol. Cell.

[62] Mann J.R., Gleixner A.M., Mauna J.C., Gomes E., DeChellis-Marks M.R., Needham P.G., Copley K.E., Hurtle B., Portz B., Pyles N.J. (2019). RNA Binding Antagonizes Neurotoxic Phase Transitions of TDP-43. Neuron.

[63] Cooper-Knock J., Higginbottom A., Stopford M.J., Highley J.R., Ince P.G., Wharton S.B., Pickering-Brown S., Kirby J., Hautbergue G.M., Shaw P.J. (2015). Antisense RNA foci in the motor neurons of C9ORF72-ALS patients are associated with TDP-43 proteinopathy. Acta Neuropathol..

[64] Zhang Y.J., Jansen-West K., Xu Y.F., Gendron T.F., Bieniek K.F., Lin W.L., Sasaguri H., Caulfield T., Hubbard J., Daughrity L. (2014). Aggregation-prone c9FTD/ALS poly(GA) RAN-translated proteins cause neurotoxicity by inducing ER stress. Acta Neuropathol..

[65] Narcis J.O., Tapia O., Tarabal O., Piedrafita L., Caldero J., Berciano M.T., Lafarga M. (2018). Accumulation of poly(A) RNA in nuclear granules enriched in Sam68 in motor neurons from the SMNDelta7 mouse model of SMA. Sci. Rep..

[66] Kukharsky M.S., Quintiero A., Matsumoto T., Matsukawa K., An H., Hashimoto T., Iwatsubo T., Buchman V.L., Shelkovnikova T.A. (2015). Calcium-responsive transactivator (CREST) protein shares a set of structural and functional traits with other proteins associated with amyotrophic lateral sclerosis. Mol. Neurodegener..

[67] Owens M.C., Shen H., Yanas A., Mendoza-Figueroa M.S., Lavorando E., Wei X., Shweta H., Tang H.Y., Goldman Y.E., Liu K.F. (2023). Mutant forms of DDX3X with diminished catalysis form hollow condensates that exhibit sex-specific regulation. bioRxiv.

[68] Grese Z.R., Bastos A.C., Mamede L.D., French R.L., Miller T.M., Ayala Y.M. (2021). Specific RNA interactions promote TDP-43 multivalent phase separation and maintain liquid properties. EMBO Rep..

[69] Shelkovnikova T.A., Robinson H.K., Southcombe J.A., Ninkina N., Buchman V.L. (2014). Multistep process of FUS aggregation in the cell cytoplasm involves RNA-dependent and RNA-independent mechanisms. Hum. Mol. Genet..

[70] Maharana S., Wang J., Papadopoulos D.K., Richter D., Pozniakovsky A., Poser I., Bickle M., Rizk S., Guillén-Boixet J., Franzmann T.M. (2018). RNA buffers the phase separation behavior of prion-like RNA binding proteins. Science.

[71] Tank E.M., Figueroa-Romero C., Hinder L.M., Bedi K., Archbold H.C., Li X., Weskamp K., Safren N., Paez-Colasante X., Pacut C. (2018). Abnormal RNA stability in amyotrophic lateral sclerosis. Nat. Commun..

[72] Spence H., Waldron F.M., Saleeb R.S., Brown A.L., Rifai O.M., Gilodi M., Read F., Roberts K., Milne G., Wilkinson D. (2024). RNA aptamer reveals nuclear TDP-43 pathology is an early aggregation event that coincides with STMN-2 cryptic splicing and precedes clinical manifestation in ALS. Acta Neuropathol..

[73] Hutten S., Usluer S., Bourgeois B., Simonetti F., Odeh H.M., Fare C.M., Czuppa M., Hruska-Plochan M., Hofweber M., Polymenidou M. (2020). Nuclear Import Receptors Directly Bind to Arginine-Rich Dipeptide Repeat Proteins and Suppress Their Pathological Interactions. Cell Rep..

[74] Al-Sarraj S., King A., Troakes C., Smith B., Maekawa S., Bodi I., Rogelj B., Al-Chalabi A., Hortobágyi T., Shaw C.E. (2011). p62 positive, TDP-43 negative, neuronal cytoplasmic and intranuclear inclusions in the cerebellum and hippocampus define the pathology of C9orf72-linked FTLD and MND/ALS. Acta Neuropathol..

[75] Vatsavayai S.C., Yoon S.J., Gardner R.C., Gendron T.F., Vargas J.N.S., Trujillo A., Pribadi M., Phillips J.J., Gaus S.E., Hixson J.D. (2016). Timing and significance of pathological features in C9orf72 expansion-associated frontotemporal dementia. Brain.

[76] Baborie A., Griffiths T.D., Jaros E., Perry R., McKeith I.G., Burn D.J., Masuda-Suzukake M., Hasegawa M., Rollinson S., Pickering-Brown S. (2015). Accumulation of dipeptide repeat proteins predates that of TDP-43 in frontotemporal lobar degeneration associated with hexanucleotide repeat expansions in C9ORF72 gene. Neuropathol. Appl. Neurobiol..

[77] Yan X., Kuster D., Mohanty P., Nijssen J., Pombo-García K., Rizuan A., Franzmann T.M., Sergeeva A., Passos P.M., George L. (2024). Intra-condensate demixing of TDP-43 inside stress granules generates pathological aggregates. bioRxiv.

[78] West R.J.H., Sharpe J.L., Voelzmann A., Munro A.L., Hahn I., Baines R.A., Pickering-Brown S. (2020). Co-expression of C9orf72 related dipeptide-repeats over 1000 repeat units reveals age- and combination-specific phenotypic profiles in Drosophila. Acta Neuropathol. Commun..

[79] Miyagi T., Ueda K., Sugimoto M., Yagi T., Ito D., Yamazaki R., Narumi S., Hayamizu Y., Uji-I H., Kuroda M., Kanekura K. (2023). Differential toxicity and localization of arginine-rich C9ORF72 dipeptide repeat proteins depend on de-clustering of positive charges. iScience.

[80] Shelkovnikova T.A., Robinson H.K., Troakes C., Ninkina N., Buchman V.L. (2014). Compromised paraspeckle formation as a pathogenic factor in FUSopathies. Hum. Mol. Genet..

[81] Lee M., Sadowska A., Bekere I., Ho D., Gully B.S., Lu Y., Iyer K.S., Trewhella J., Fox A.H., Bond C.S. (2015). The structure of human SFPQ reveals a coiled-coil mediated polymer essential for functional aggregation in gene regulation. Nucleic Acids Res..

[82] Bauer C.S., Webster C.P., Shaw A.C., Kok J.R., Castelli L.M., Lin Y.H., Smith E.F., Illanes-Álvarez F., Higginbottom A., Shaw P.J. (2022). Loss of TMEM106B exacerbates C9ALS/FTD DPR pathology by disrupting autophagosome maturation. Front. Cell. Neurosci..

[83] Webster C.P., Smith E.F., Bauer C.S., Moller A., Hautbergue G.M., Ferraiuolo L., Myszczynska M.A., Higginbottom A., Walsh M.J., Whitworth A.J. (2016). The C9orf72 protein interacts with Rab1a and the ULK1 complex to regulate initiation of autophagy. EMBO J..

[84] Shelkovnikova T.A., Kukharsky M.S., An H., Dimasi P., Alexeeva S., Shabir O., Heath P.R., Buchman V.L. (2018). Protective paraspeckle hyper-assembly downstream of TDP-43 loss of function in amyotrophic lateral sclerosis. Mol. Neurodegener..

